# Fluorination as a Molecular Design Parameter for Programming Peptide Nanocarriers

**DOI:** 10.1002/smll.74993

**Published:** 2026-08-02

**Authors:** Maurizio Iannuzzi, Holly Fiedler, Kilian Haoues, Suvrat Chowdhary, Hossein Batebi, Anil Kumar Sahoo, Andreas F. Thünemann, Boris Schade, Elisa Quaas, Roland R. Netz, Beate Koksch

**Affiliations:** ^1^ Department of Chemistry and Biochemistry Freie Universität Berlin Berlin Germany; ^2^ Department of Physics Freie Universität Berlin Berlin Germany; ^3^ Federal Institute for Materials Research and Testing (BAM) Berlin Germany; ^4^ Center for Electron Microscopy Research (FZEM) Institute of Chemistry and Biochemistry Freie Universität Berlin Berlin Germany; ^5^ Institute of Chemistry and Biochemistry and Core Facility SupraFAB Freie Universität Berlin Berlin Germany

**Keywords:** biocompatible materials, fluorinated amino acids, fluoropeptides, supramolecular nanocarriers, targeted drug delivery

## Abstract

Peptide‐based nanocarriers offer unique opportunities for programmable self‐assembly (SA), yet rational design strategies that couple structural control with biological function remain limited. In particular, fluoropeptides have been largely inaccessible as modular building blocks until the recent availability of enantiomerically pure fluorinated amino acids (FAAs) in gram‐scale quantities, preventing their systematic use in functional nanomaterials. Here, we introduce fluorination as a quantitative molecular design parameter to program peptide SA, morphology, and biological performance. A homologous series of amphiphilic fluoropeptide–RGD conjugates was constructed in which the degree of side‐chain fluorination is precisely controlled. We show that fluorination does not monotonically enhance assembly, but instead defines a narrow design window in which compact, non‐amyloid fibrillar architectures emerge. Mechanistic insight from molecular dynamics (MD) simulations reveals how fluorine‐induced modulation of hydrophobic interactions and hydration disrupts classical β‐sheet packing. The optimized trifluorinated conjugate exhibits high drug‐loading capacity, pH‐triggered release, receptor‐specific cellular uptake, and low cytotoxicity, while remaining enzymatically degradable. Together, these results establish fluorinated peptide segments as a previously inaccessible and programmable handle for the rational design of peptide‐based nanocarriers.

## Graphical abstract

Controlled side‐chain fluorination programs the SA, morphology, and biological performance of amphiphilic fluoropeptide–RGD nanocarriers. A narrow fluorination window yields compact non‐amyloid fibrils with high loading capacity, pH‐triggered release, receptor‐specific uptake, low cytotoxicity, and enzymatic degradability.

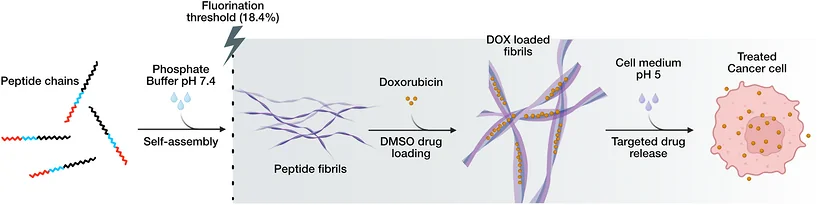

## 1 Introduction

Peptides provide a versatile platform for the construction of functional nanomaterials, as their primary sequence can be programmed to encode self‐assembly, morphology, and biological activity. This has enabled the development of supramolecular peptide architectures for applications ranging from biomaterials to drug delivery. However, translating peptide SA into functional nanocarriers remains challenging, as small changes in molecular interactions can shift assemblies from dynamic, hydrated nanostructures, which are generally preferred for drug delivery due to their capacity for reversible cargo encapsulation and release and their adaptability in biological environments [1, 2]. In contrast, rigid, amyloid‐like fibrils exhibit dense molecular packing and high kinetic stability, often resulting in poor loading efficiency and limited release, thereby restricting their utility in this field [3].

Identifying molecular design parameters that reliably control peptide assembly pathways while preserving biological compatibility therefore remains a central challenge [4, 5, 6, 7, 8, 9, 10].

Chemical modification of amino acid side chains represents a powerful strategy to expand the functional space of peptide‐based materials. Among these approaches, fluorination is particularly attractive, as fluorine substitution introduces localized polarity into otherwise hydrophobic side chains, thereby modulating hydrophobic interactions, packing behavior, and hydration [11, 12, 13, 14]. While fluorine substituents are widely employed in small‐molecule drug design, their systematic incorporation into peptide architectures has remained largely unexplored. This limitation has been primarily synthetic in nature, as enantiomerically pure fluorinated amino acid building blocks have only recently become available in quantities compatible with peptide synthesis and materials‐oriented studies [15, 16]. As a consequence, fluorinated peptides have not yet been established as a general and modular design element for programming peptide SA and function. Whether side‐chain fluorination can be exploited as a quantitative molecular parameter to control peptide assembly pathways, rather than merely perturbing them, has therefore remained an open question.

Advances in peptide and protein conjugation chemistry have enabled the design of fluorinated amphiphiles and polymeric nanocarriers that promote efficient cargo encapsulation and intracellular delivery [17]. Such systems highlight how strategic fluorination can modulate molecular interactions with membranes and biological environments to improve delivery efficacy [18, 19]. Here, by applying rational design principles, we developed a novel polyfluorinated delivery system that combines the stability and membrane permeability of fluoropolymers with the precision and biocompatibility of peptides.

Building on the recent synthetic accessibility of FAA building blocks, we set out to exploit side‐chain fluorination as a rational design parameter for programming peptide SA and function [20, 21]. We have screened a series of amphiphilic fluoropeptide–RGD conjugates in which the degree of fluorination within the hydrophobic segment is systematically varied, while the overall peptide architecture and bioactive motif are kept constant to systematically explore how variations in hydrophobic core length and degree of fluorination govern SA. This approach enables us to delineate the structural threshold required for assembly and to elucidate potential structure‐function correlations. This modular design further enables a direct correlation between fluorine content, supramolecular organization, and biological performance. By combining a fluorinated peptide segment with an integrin‐binding RGD motif, we aim to couple controlled self‐assembly with receptor‐specific cellular uptake and pH‐responsive drug release (Figure 1). RGD‐derived sequences target integrin receptors, which are overexpressed on various tumor cells and angiogenic endothelial cells, enabling selective accumulation in tumor‐associated tissues. Following integrin‐mediated binding, the assemblies are expected to undergo receptor‐mediated endocytosis. The resulting decrease in pH in endosomal environments provides a trigger for drug release, for which doxorubicin was used as a model drug. Using this platform, we examine how incremental increases in fluorine content govern peptide hydrophobicity, secondary structure formation, and supramolecular morphology, and identify a narrow fluorination window that enables efficient drug encapsulation without inducing amyloid‐like aggregation.

**FIGURE 1:**
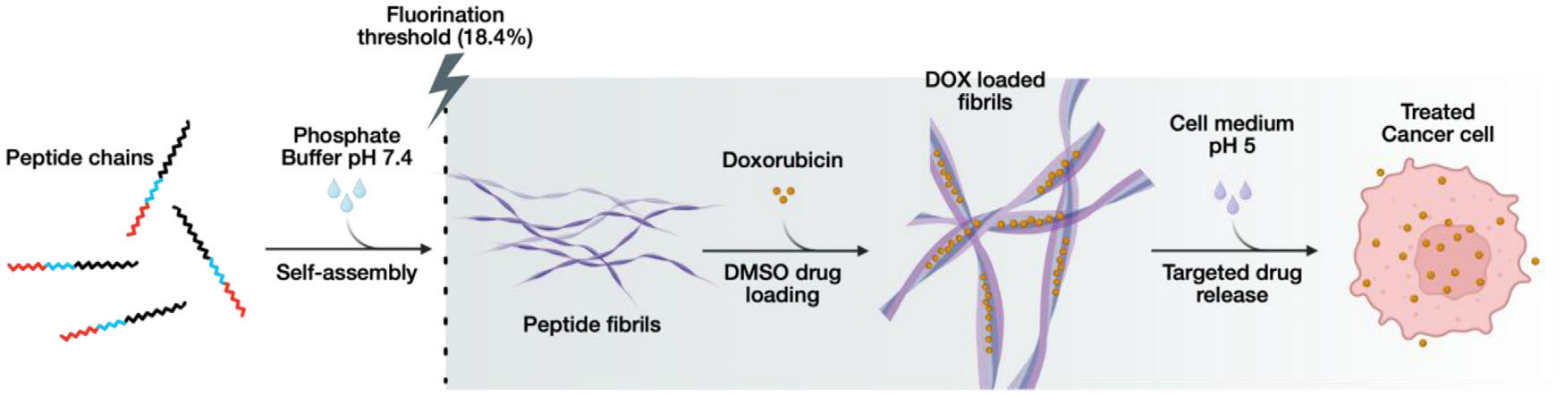
Fluorination as a molecular design parameter for programming peptide self‐assembly and function.

## 2 Results and Discussion

### 2.1 Rational Design and Synthesis of Amphiphilic Fluoropeptide Nanocarriers

To translate side‐chain fluorination into a programmable design parameter, we designed an amphiphilic co‐block peptide architecture in which a fluorinated peptide segment drives SA, while a C‐terminal RGD motif provides biological functionality (Figure 2A) [22]. The modular peptide architecture follows the general sequence X_4‐8_ –GY–GKK–GRGDS, in which the N‐terminal X_4‐8_ segment constitutes the fluorinated hydrophobic block of 4–8 units of FAAs or the non‐fluorinated control, respectively, while the C‐terminal GKK–GRGDS motif provides solubility, pH responsiveness, and integrin‐specific targeting. A homologous library of fluoropeptide–RGD conjugates was constructed by systematically varying the degree of side‐chain fluorination within the hydrophobic segment while keeping the overall peptide architecture constant (Table S2). Valine (Val) was used as a non‐fluorinated reference, and a series of FAAs with increasing fluorine content served as modular building blocks.

**FIGURE 2:**
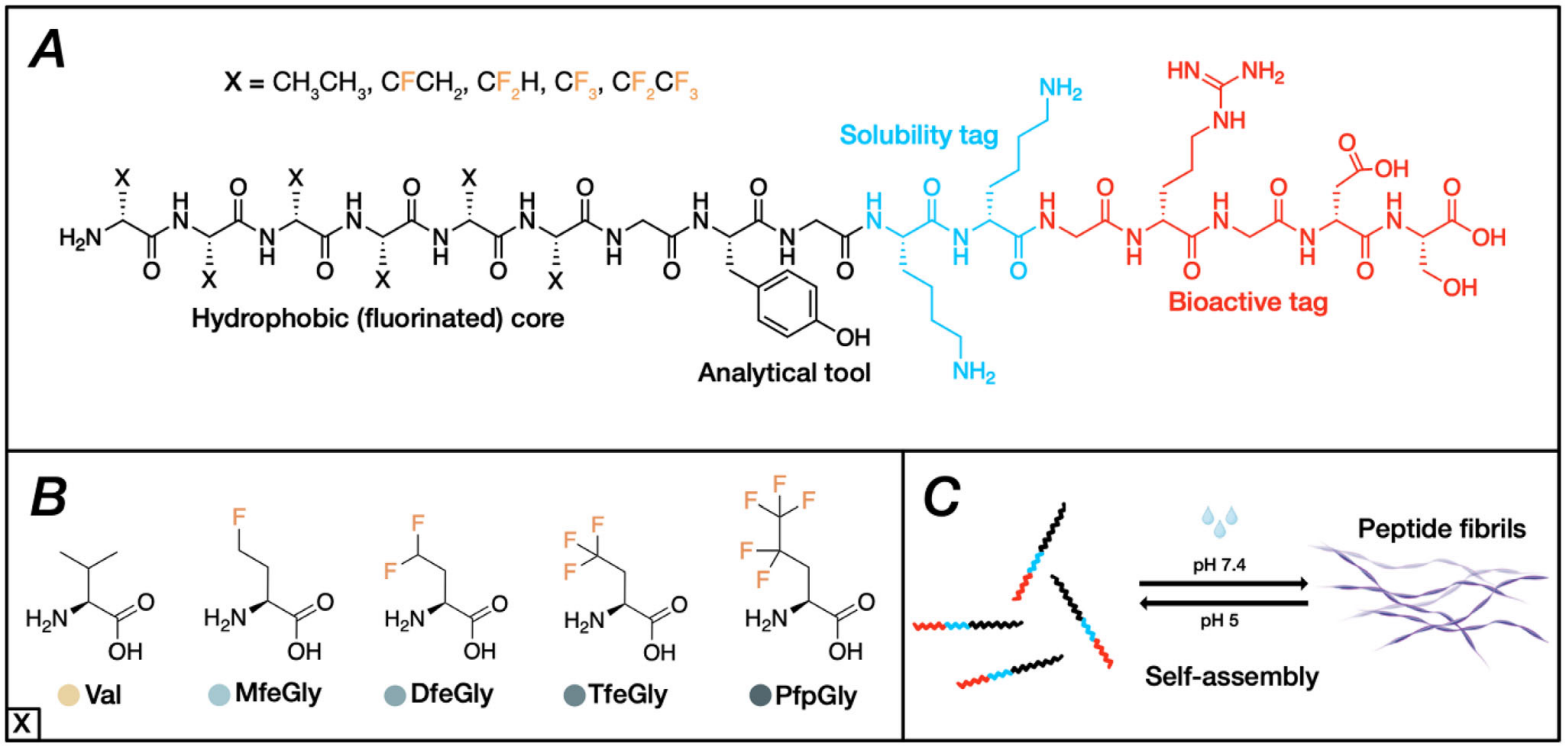
(A) Peptide design Val6RGD, MfeGly6RGD, DfeGly6RGD, TfeGly6RGD and PfpGly6RGD. (B) Chemical structures of amino acids Val, MfeGly, DfeGly, TfeGly, and PfpGly. (C) Schematic illustration showing the pH‐responsive self‐assembly mechanism.

This approach enables a direct comparison of fluorination‐dependent effects on peptide hydrophobicity, secondary structure formation, and supramolecular assembly. In the following, “short” hydrophobic segments refer to conjugates containing four hydrophobic residues, whereas “extended” segments comprise six or more hydrophobic residues within the N‐terminal block. All conjugates were prepared by solid‐phase peptide synthesis (Table S1) and purified to analytical purity. While shorter hydrophobic segments were readily accessible, increasing the length and fluorine content of the fluoropeptide block led to progressively more challenging syntheses, consistent with an enhanced tendency toward aggregation during assembly on the solid support. With this modular architecture in place, we next examined how systematic variation of side‐chain fluorination modulates the intrinsic hydrophobicity of the conjugates, as a first descriptor governing peptide self‐assembly.

### 2.2 Library Screening and Hydrophobicity of Fluoropeptide Conjugates

To identify the minimum hydrophobic segment length required for stable SA, a series of peptide amphiphiles with systematically varied hydrophobic domains in terms of number and fluorination degree of individual hydrophobic amino acid building blocks was synthesized. CD spectroscopy was used to evaluate the emergence of ordered secondary structure associated with possible assembly, whereas cryo‐TEM provided direct evidence of nanostructure formation. The results of these screenings highlight that short hydrophobic segments were sufficient to induce weak secondary‐structure signatures but did not yield persistent supramolecular assemblies (Figure S18). In contrast, peptide sequences containing extended hydrophobic blocks of six amino acids led to efficient self‐assembly. Herein, we further synthesized the polyfluorinated amphiphilic hexameric oligopeptides MfeGly6RGD (NH_2_‐MfeGly_6_GYGKKGRGDS‐OH), DfeGly6RGD (NH_2_‐DfeGly_6_GYGKKGRGDS‐OH) and PfpGly6RGD (NH_2_‐PfpGly_6_GYGKKGRGDS‐OH) by substitution of Val with the respective FAAs monofluoroethylglycine (MfeGly), difluoroethylglycine (DfeGly) and pentafluoropropylglycine (MfeGly). However, the synthesis of the longer octameric peptide conjugates proved significantly more challenging. Pronounced aggregation on the solid‐phase resin resulted in markedly low yields, with only trace amounts recovered after purification (Table S2).

To establish side‐chain fluorination as a quantitative design parameter, we first evaluated how incremental fluorine substitution modulates the intrinsic hydrophobicity of the peptide conjugates (Figure 3). Using RP‐HPLC retention as an experimental proxy, hydrophobicity was found to increase with incremental side‐chain fluorination. Notably, this trend was non‐monotonic: incorporation of a single fluorine atom (MfeGly) resulted in reduced retention relative to the non‐fluorinated control, consistent with an increase in side‐chain polarity.

**FIGURE 3:**
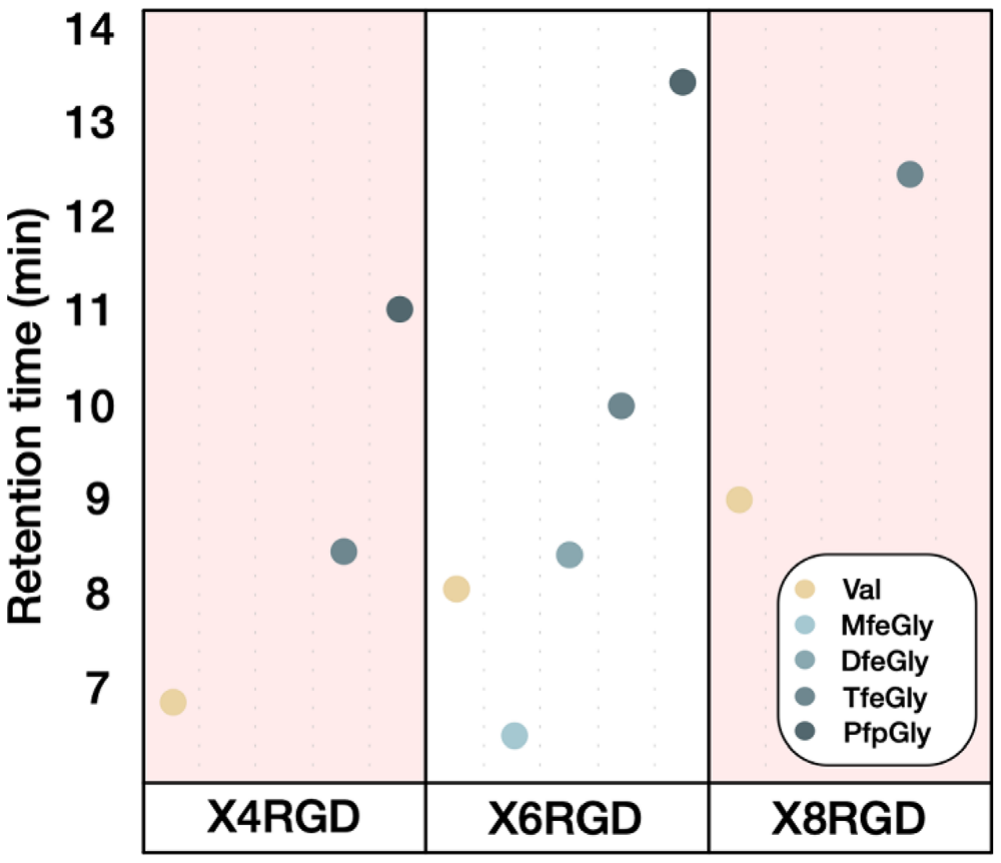
Retention times of the fluoropeptide conjugates in comparison to the non‐fluorinated control as an experimental index of intrinsic hydrophobicity. An increase in retention time corresponds to a stronger hydrophobic character.

These results demonstrate that fluorination does not act as a simple hydrophobicity amplifier. In addition to increasing hydrophobic surface area at higher substitution levels, fluorine exerts a strong inductive effect that enhances side‐chain polarity and modulates local dipole moments. This is particularly evident for monofluorinated residues (MfeGly), where the increased side‐chain polarity and hydration outweigh hydrophobic contributions, resulting in reduced retention time, consistent with our previous findings [20]. At higher degrees of fluorination, however, the cumulative effect of increased surface area and reduced polarizability promotes hydrophobic interactions.

Thus, the observed non‐monotonic trend arises from a balance between fluorine‐induced polarity, dipole interactions, and hydrophobic surface area, rather than hydrophobicity alone. These electronic effects are expected to influence not only solvation but also intermolecular interactions, including backbone hydrogen bonding and side‐chain packing, which subsequently govern secondary structure formation and supramolecular assembly.

In view of the lack of self‐assembly observed for the shorter sequences and the complications associated with longer ones, only the hexameric sequence and its fluoropeptide variants with fluorine content between 7% and 26.4% were chosen for further characterization.

### 2.3 Secondary Structure Determination, Self‐Assembly and pH Responsiveness

To determine how fluorination‐dependent hydrophobicity translates into peptide folding and assembly, we analyzed secondary structure formation and pH responsiveness under conditions that support stable yet soluble assemblies (Figure 4). Although peptide SA was already observed at concentrations as low as 0.1 wt.% (500 µM), structural characterization was performed at 1 wt.% to ensure consistency across all methods and conditions and to enable direct correlation between structural data.

**FIGURE 4:**
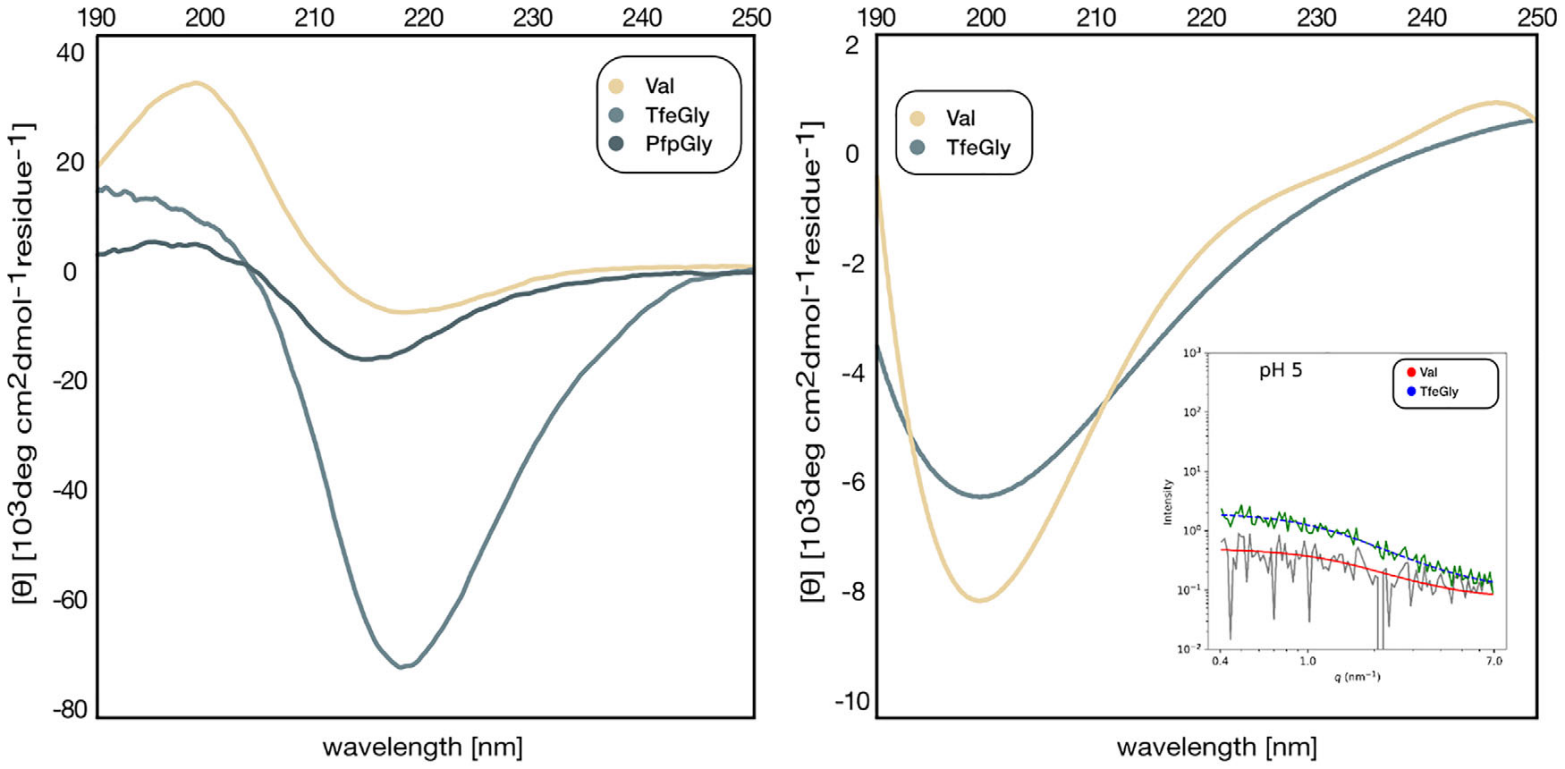
CD spectra of amphiphilic oligopeptides Val6RGD, TfeGly6RGD and PfpGly6RGD at pH 7.4 (left) and of Val6RGD and TfeGly6RGD at pH 5.0 (right). The absence of self‐assembled structures was confirmed by SAXS measurements (bottom right).

At physiological pH (7.4), the non‐fluorinated reference Val6RGD adopted a β‐sheet‐rich conformation consistent with amyloid‐like assembly (Figure 4). Fluorinated variants showed markedly different responses: monofluorination favored disordered conformations (Figure S13), while higher degrees of fluorination promoted non‐canonical signatures accompanied by time‐dependent spectral changes indicative of supramolecular reorganization and different degrees of order (Figure S14). These observations suggest that fluorination alters the balance between β‐sheet propensity and hydration, thereby redirecting folding and assembly pathways, suppressing β‐sheet formation.

Because pH responsiveness is central to drug‐release functionality, assemblies were further examined under mildly acidic conditions (pH 5). Under these conditions, both Val6RGD and the fluorinated conjugate TfeGly6RGD displayed a loss of ordered secondary structure, consistent with electrostatic repulsion arising from N‐terminal amine protonation confirmed via MD simulations (Figure S23). Small‐angle X‐ray scattering (SAXS) measurements under acidic conditions supported the absence of assembled structures and were best described by random‐coil models, demonstrating reversible pH‐triggered disassembly (Figure 4). Having established fluorination‐ and pH‐dependent folding behavior, we next investigated how these differences manifest in supramolecular morphology.

### 2.4 Morphology of Self‐Assembled Conjugates

To elucidate how fluorination‐dependent folding behavior translates into supramolecular organization, we examined the morphology of the assembled hexameric conjugates under physiological conditions (Figure 5A).

**FIGURE 5:**
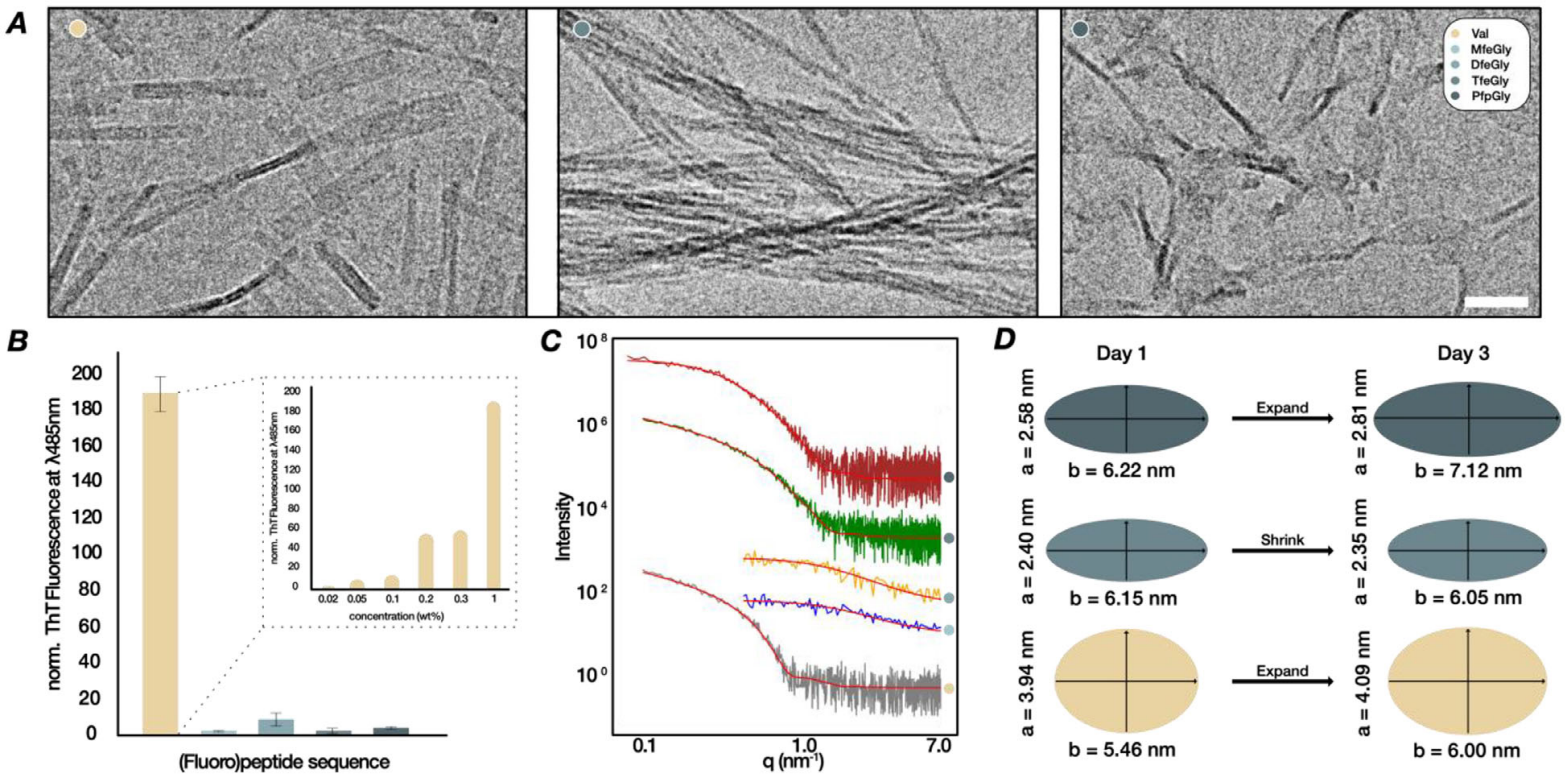
(A) Cryo‐TEM micrographs of Val6RGD (left), TfeGly6RGD (center), and PfpGly6RGD (right) illustrate the effect of increasing degrees of fluorination on self‐assembly. The scale bar corresponds to 100 nm. (B) ThT assays of Val6RGD‐PfpGly6RGD; fluorescence emission confirms amyloid formation exclusively for Val6RGD. Data are reported as Mean ± SE, *n* = 3. (C) SAXS profiles of amphiphilic oligopeptides at pH 7.4 and (D) Fibril analysis of the elliptical cross‐sections of Val6RGD, TfeGly6RGD, and PfpGly6RGD reveal different packing organizations.

Cryogenic transmission electron microscopy (Cryo‐TEM) revealed pronounced differences across the series. The non‐fluorinated reference Val6RGD formed rigid, platelet‐like fibrillar structures characteristic of amyloid‐type assemblies. In contrast, screened conjugates bearing short hydrophobic segments, as well as mono‐ and difluorinated conjugates (Figure S18), showed no detectable supramolecular structures, consistent with their partially disordered secondary‐structure signatures (Figure S13).

Strikingly, conjugates containing extended fluorinated hexameric segments exhibited distinct, non‐amyloid morphologies. The trifluorinated system assembled into flexible structures that further associated into regular bundles, indicative of a hierarchical organization. Increasing steric demand through higher fluorination (PfpGly) altered the packing behavior and led to the formation of morphologies that deviated from classical fibrillar architectures. These observations demonstrate that side‐chain fluorination not only modulates the propensity for peptide SA, but also reprograms supramolecular morphology, suppressing rigid amyloid‐like structures in favor of compact yet flexible assemblies.

To independently assess the amyloidogenic character of the assemblies, we employed thioflavin T (ThT) fluorescence spectroscopy (Figure 5B). This dye exhibits a marked increase in fluorescence emission (λ_em_ = 485 nm) upon binding to the ordered cross‐β‐sheet‐rich structures of amyloid fibrils, due to restriction of intramolecular rotation in a rigid binding environment [23]. Accordingly, ThT fluorescence is here used as an indicator of amyloid formation. Across a broad concentration range, none of the fluorinated conjugates exhibited a significant ThT response, indicating the absence of cross‐β amyloid structures, according to CD analysis. In contrast, the non‐fluorinated β‐sheet‐inducing reference Val6RGD displayed a pronounced, time‐ and concentration‐dependent increase in ThT fluorescence, consistent with the formation of amyloid‐like fibrils and clear β‐sheet conformation (Figure S22).

These results corroborate the cryo‐TEM observations and demonstrate that side‐chain fluorination effectively suppresses amyloid formation, even under conditions where the non‐fluorinated sequence readily adopts cross‐β architectures. ThT thus provides an orthogonal validation that the fluorinated assemblies represent structurally and mechanistically distinct supramolecular assemblies rather than modified amyloid fibrils.

Having defined that the fluorinated assemblies are non‐amyloid in nature, we next sought to elucidate how fluorination alters the molecular packing and internal organization of the supramolecular structures. SAXS was employed to probe the internal packing and global dimensions of the assembled conjugates under physiological conditions. The scattering profiles of the non‐fluorinated reference Val6RGD were consistent with elongated filamentous structures featuring an elliptical cross‐section, in agreement with amyloid‐like fibrillar morphologies observed by cryo‐TEM (Figure 5C). In contrast, fluorinated conjugates that formed supramolecular assemblies exhibited markedly different packing characteristics. The optimized trifluorinated conjugate was best described by a filamentous model with a reduced cross‐sectional area, indicative of more compact molecular organization. Increasing fluorination further altered the cross‐sectional anisotropy, reflecting changes in lateral packing and steric constraints imposed by the fluorinated side chains.

Comparative analysis of the fitted cross‐sections revealed that fluorinated assemblies pack more densely along the minor axis than the non‐fluorinated reference, consistent with a reorganization of intermolecular contacts driven by fluorine‐specific interactions (Figure 5D). These packing differences correlate with the suppression of amyloid‐like fibril formation and the emergence of flexible, non‐amyloid supramolecular architectures.

Time‐resolved SAXS measurements further showed that fluorinated assemblies reached stable cross‐sectional dimensions within experimental uncertainty, whereas the non‐fluorinated system continued to evolve over time, suggesting slower structural relaxation toward optimal packing.

Together, these results identify fluorination as a key determinant of supramolecular packing, governing both the internal organization and temporal stability of peptide assemblies.

### 2.5 Carrier Properties and Core–Shell Assemblies

The modular co‐block architecture of the peptide conjugates resembles that of classical amphiphilic systems, which can self‐assemble into core–shell nanostructures featuring a hydrophobic interior and a hydrophilic exterior. To evaluate whether the assembled conjugates form such compartmentalized architectures capable of encapsulating hydrophobic cargos, we employed pyrene fluorescence as a sensitive probe for hydrophobic microenvironments.

At peptide concentrations below 0.1 wt.%, no significant pyrene fluorescence was detected, indicating the absence of defined hydrophobic domains (Figure S30). In contrast, from 0.1 wt.% concentration, a dramatic increase in fluorescence intensity was observed for the non‐fluorinated Val6RGD and the trifluorinated TfeGly6RGD, consistent with the formation of core–shell assemblies capable of solubilizing pyrene within a hydrophobic interior. Both systems exhibited their highest encapsulation efficiency at elevated peptide concentrations of 1 wt.% (Figure 6). Consistent with the other structural data, the packing patterns of MfeGly and DfeGly conjugates do not support SA, and accordingly no pyrene fluorescence was detected. By comparison, the PfpGly conjugate containing more sterically demanding fluorinated side chains failed to promote efficient pyrene encapsulation, indicating that not all assembled morphologies support the formation of well‐defined hydrophobic cores.

**FIGURE 6:**
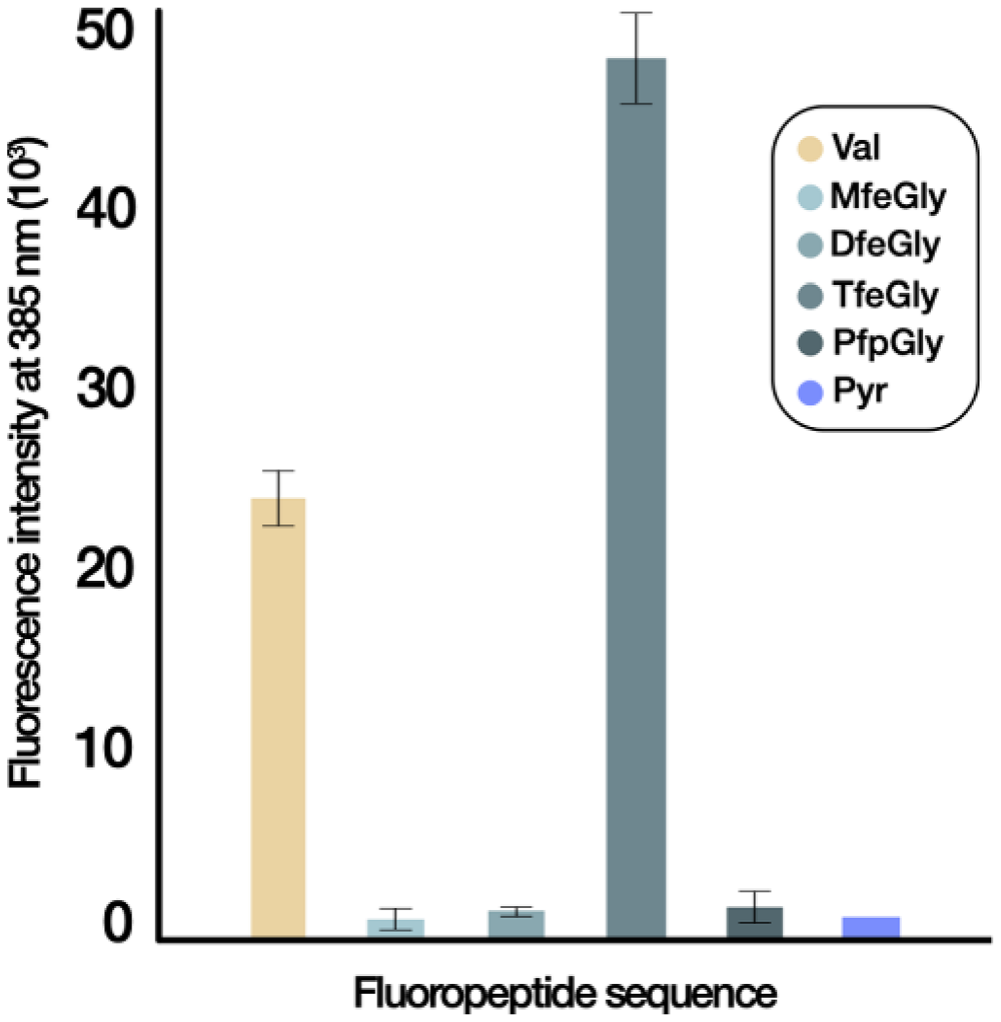
FL‐based investigation of (fluoro)peptides after addition of water‐insoluble probe pyrene. An enhancement in fluorescence intensity indicates the encapsulation and solubilization of pyrene in a hydrophobic inner core. Data are reported as Mean ± SE, *n* = 3.

These results identify Val6RGD and TfeGly6RGD as the only members of the investigated series that combine stable self‐assembly with effective core–shell compartmentalization. However, the structural differences between these conjugates are heavily reflected in their carrier properties. On this basis, these two conjugates were selected for subsequent mechanistic analysis and functional evaluation as pH‐responsive peptide‐based carriers. To rationalize why only selected assemblies combine structural integrity with carrier functionality, we next turned to all‐atom simulations.

### 2.6 Molecular Dynamics Simulations: Investigating Fluorine‐Specific Interactions

To elucidate the molecular origin of the fluorination‐dependent assembly pathways observed experimentally, we performed MD simulations on representative peptide systems under conditions that mirror the experimental environment. Initial assessment of protonation states identified the N‐terminal amine deprotonation as a key determinant governing assembly formation under neutral medium pH conditions according to experimental results (Figure S23). Simulations underline how Val6RGD rapidly adopted stable β‐sheet structures, while TfeGly6RGD remained largely disordered throughout (Figure 7). However, TfeGly6RGD still forms compact supramolecular aggregates. To elucidate the molecular mechanism underlying the fluorine‐induced β‐sheet disruption and structural compaction, inter‐chain contacts (Figure S26) were studied. Detailed contact analysis shows that TfeGly substitution altered the balance between backbone and side‐chain interactions. Whereas Val6RGD systems exhibited frequent backbone–side chain and side chain–side chain contacts, TfeGly6RGD assemblies displayed markedly fewer side‐chain interactions. Instead, stabilization relied more heavily on backbone–backbone contacts (Figure S27).

**FIGURE 7:**
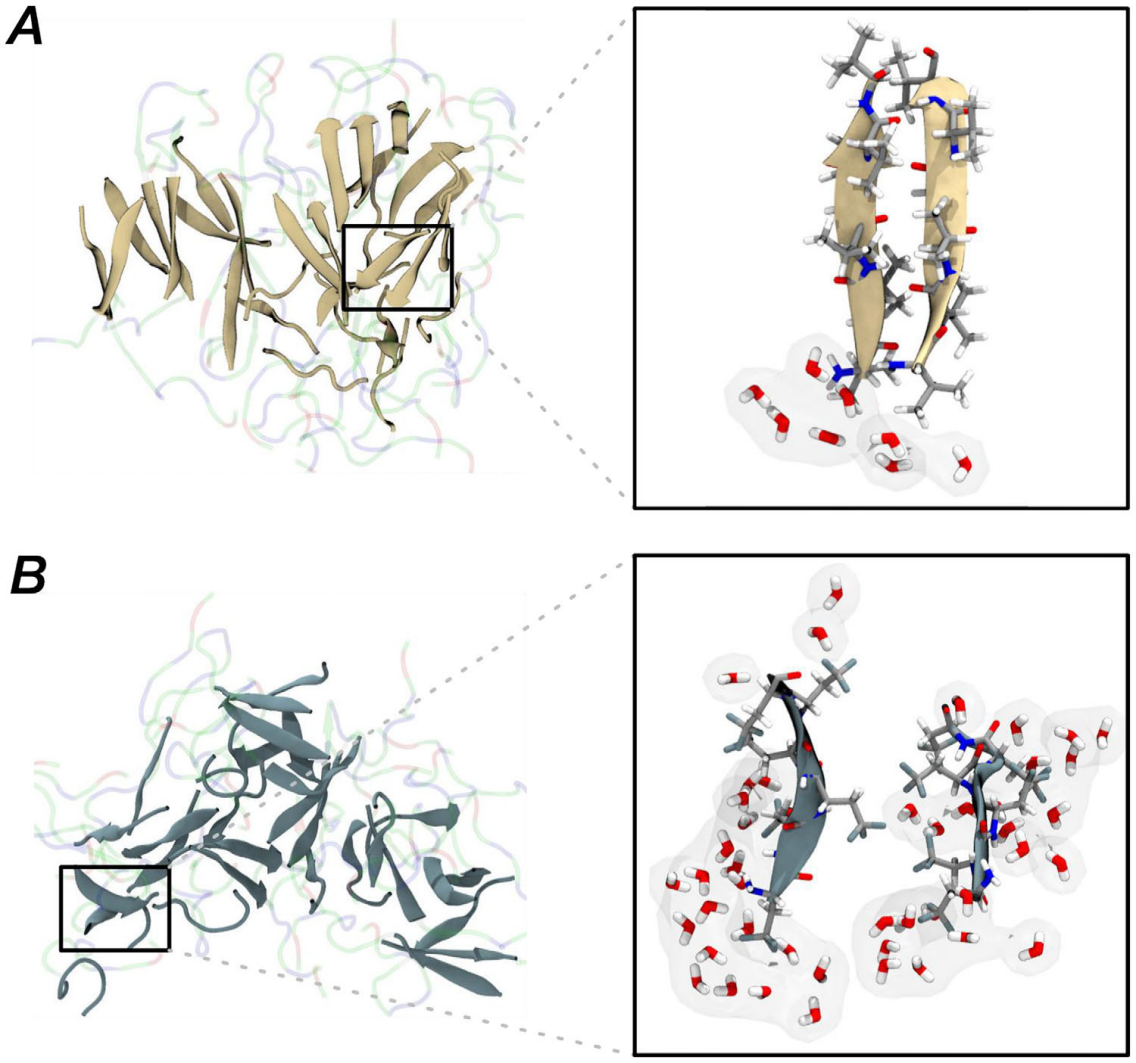
Representative snapshots from the end of a 2 µs MD trajectory showing peptide assemblies formed by (A) Val6RGD and (B) TfeGly6RGD. The Val6RGD system forms ordered β‐sheet‐rich assemblies, visible as aligned peptide strands arranged in sheet‐like structures. In contrast, the fluorinated TfeGly6RGD system exhibits more disordered assemblies, where peptide strands adopt irregular orientations and lack extended β‐sheet organization. The zoomed views of the boxed regions show the atomistic peptide backbone arrangement and the nearby water molecules located within 5 Å of the peptide backbone. For the Val6RGD system, peptides are tightly packed with backbone contacts consistent with β‐sheet formation and reduced hydration. In contrast, the TfeGly6RGD system shows a more hydrated and less ordered structure.

Compared with the non‐fluorinated Val6RGD system, fluorine substitution weakens side‐chain–side‐chain contacts while preserving backbone interactions, resulting in assemblies that remain densely packed but are more hydrated (Figure 7B), leading to more flexible and dynamic fibrillar assemblies (Figure 5A). This mechanistic difference is visible in the zoomed views in Figure 7, where the fluorinated TfeGly6RGD assembly shows more water molecules surrounding the peptide backbones than Val6RGD. Hydration analysis confirms this trend, revealing a higher number of water molecules within 5 Å of the peptide backbones in TfeGly6RGD (Figure S28). At the same time, backbone association persists without forming cooperative cross‐β packing (Figure S26). The resulting aggregates remain structurally stable but do not adopt amyloid‐like organization, instead retaining higher internal hydration (Figure S28). This behavior is consistent with the flexible fibrillar morphologies observed by cryo‐TEM and the compact cross‐sections identified by SAXS.

The simulations reveal that side‐chain fluorination reshapes the balance between backbone interactions, side‐chain contacts, and hydration rather than simply preventing aggregation. TfeGly6RGD does not form beta sheets but still forms compact supramolecular aggregates, as shown by the radius‐of‐gyration analysis, and retains increased backbone‐associated water compared with Val6RGD, where dehydration of the peptide backbone and the formation of cooperative backbone–backbone interactions lead to compact β‐sheet packing and amyloid‐like assemblies. The retained hydration of TfeGly6RGD suppresses the cooperative dehydration and directional backbone registry required for amyloid‐like cross‐β maturation, while still allowing aggregate compaction through backbone proximity and distributed peptide–peptide contacts. This interpretation is supported by the β‐sheet evolution, inter‐chain contact decomposition, residue‐level interaction, and hydration analyses shown in Figures S24–S28. To further assess the molecular packing arrangement of β‐structured regions, we classified the orientation of interchain β strand contacts in the MD trajectories (Figure S41). DSSP was used to assign β‐structured residues in each frame. Continuous β strand segments were identified for each peptide chain, and interchain β strand pairs were detected using backbone contact criteria. For each paired β strand contact, the relative orientation was determined from the N to C direction of the corresponding Cα backbone vectors and classified as parallel, antiparallel, or ambiguous. This analysis revealed that β strand contacts in Val6RGD coexist in both parallel and antiparallel orientations rather than forming a single uniform β‐sheet topology. TfeGly6RGD showed a more heterogeneous orientation distribution, supporting a model in which fluorination reduces cooperative β‐sheet packing while preserving transient backbone association. Overall, these findings demonstrate that fluorination modulates the coupling between hydration and intermolecular organization, redirecting peptide self‐assembly away from amyloid architectures toward hydrated, dynamically packed fibrillar structures. This fluorination‐induced shift in supramolecular organization is mirrored in the biological properties of the materials, including reduced amyloid‐associated cytotoxicity, improved cargo loading, and efficient pH‐triggered drug release. Taken together, the combined computational and experimental results identify fluorination as an effective strategy for tuning peptide assembly pathways and translating nanoscale structural changes into improved functional performance.

### 2.7 Enzymatic Degradation Studies

Having established the molecular mechanism by which fluorination redirects peptide SA toward hydrated, non‐amyloid architectures, we next examined how these structural features translate into enzymatic stability and biodegradability. Fluorination is frequently employed in medicinal chemistry to enhance metabolic stability; however, for peptide‐based delivery systems, enzymatic degradability remains a critical requirement for biocompatibility. We therefore investigated the susceptibility of representative hexameric conjugates, namely the non‐fluorinated reference Val6RGD and the optimized trifluorinated conjugate, toward proteolytic degradation, which was assessed under conditions where the peptides are predominantly monomeric (Figure 8). This choice was deliberate, as in their assembled state the peptides form core–shell nanostructures in which the hydrophobic segment is sequestered within the core and thus sterically shielded from enzymatic access. As a consequence, direct proteolysis of intact aggregates is expected to be significantly impeded. Instead, our aim was to evaluate the fate of the peptide following pH‐triggered disassembly and drug release, where the peptide becomes accessible to enzymatic degradation. The use of lower peptide concentrations ensured a controlled environment to probe this post‐disassembly scenario.

**FIGURE 8:**
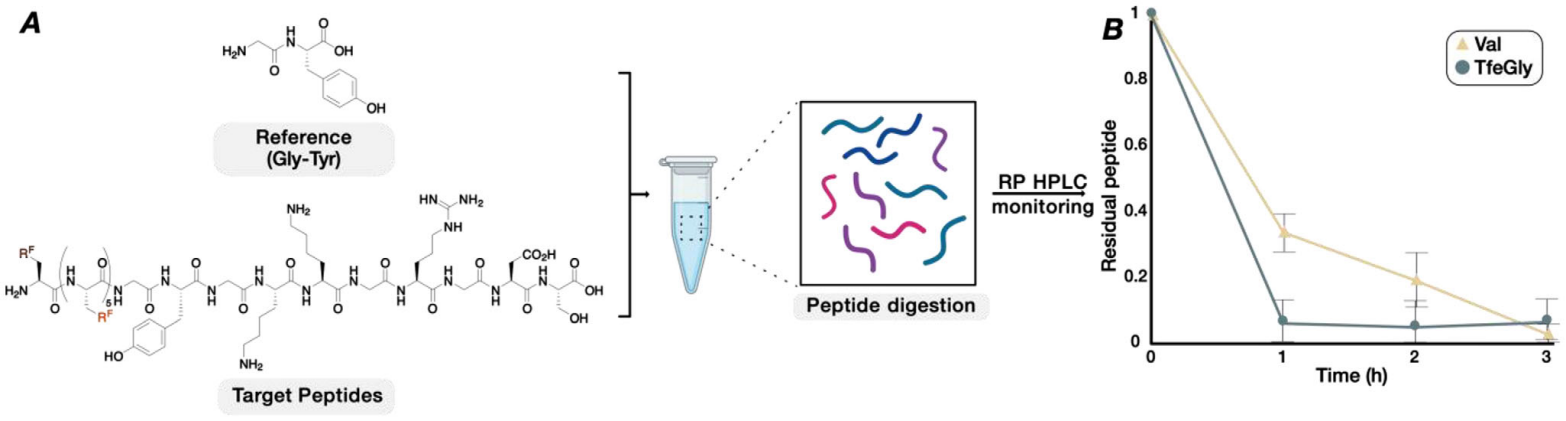
(A) Schematic illustration showing the elastase‐mediated proteolysis assay via RP‐HPLC. (B) Digestion profiles of amphiphilic oligopeptides Val6RGD and TfeGly6RGD with Elastase. Data are reported as Mean ± SE, *n* = 3.

Elastase was therefore selected as a model enzyme to specifically probe the influence of fluorinated side chains on proteolytic susceptibility while minimizing competing cleavage pathways. As a serine protease with a relatively small and hydrophobic binding pocket, elastase preferentially recognizes residues such as valine, making it particularly suitable for isolating the effect of substituting this particular amino acid with its fluorinated analogue TfeGly. In contrast, other proteases (trypsin, chymotrypsin, and Arg‐C) would target additional sites within the peptide sequence (respectively lysine, tyrosine, or arginine), thereby obscuring the contribution of the fluorinated side chains. Furthermore, elastase acts via endoproteolytic cleavage, which is expected to fragment the hydrophobic segment into smaller units, thereby reducing the potential risk of bioaccumulation associated with longer fluorinated chains.

Both conjugates were readily degraded by elastase, demonstrating that incorporation of FAAs does not confer general proteolytic resistance. Notably, TfeGly6RGD exhibited a markedly accelerated degradation profile compared to the non‐fluorinated reference. This behavior suggests that fluorination alters enzyme–substrate interactions rather than sterically shielding cleavage sites, and underscores that fluorinated assemblies remain enzymatically accessible and biodegradable, a desirable feature for peptide‐based delivery systems designed for transient therapeutic action. We attribute the enhanced degradation rate to the combined hydrophobicity and steric similarity of the trifluorinated side chains to native elastase substrates, which likely facilitates substrate recognition within the enzyme active site. These findings indicate that fluorination can increase enzymatic accessibility rather than suppress it, underscoring that fluoropeptide assemblies remain biodegradable despite their compact supramolecular organization.

While the present study demonstrates controlled degradation, several limitations should be considered when assessing the translational potential of these systems. In particular, the stability of the assemblies in physiologically relevant media remains to be established, as serum proteins, complex enzymatic environments, and dilution effects may influence supramolecular integrity, cargo retention, and cellular interactions. Although the enzymatic degradation studies indicate that TfeGly6RGD remains susceptible to degradation in the unfolded state, investigations under biologically more complex conditions will be required to assess the scope of fluorinated peptide assemblies as drug delivery platforms. Beyond enzymatic stability, safe cellular interaction is a prerequisite for functional delivery, prompting an in vitro assessment of cytotoxicity.

### 2.8 Cellular Toxicity of Fluoropeptide Conjugates

Cell studies as well as in vitro drug release experiments were then conducted at 0.1 wt.%, as this concentration is sufficient to induce SA and enable efficient cargo properties, while remaining compatible with biological assay conditions.

To assess the cellular compatibility of the peptide assemblies, we evaluated the cytotoxic effects of the entire library of peptide conjugates using a live/dead assay on A549 cells. Cells were exposed to increasing peptide concentrations under conditions that either suppress or promote supramolecular assembly for 24 h. Across the concentration range below the self‐assembly threshold, all conjugates exhibited negligible cytotoxicity, independent of fluorine content (Figures S32 and S33). Upon reaching concentrations at which assembly occurs, clear differences emerged across the series. The non‐fluorinated reference Val6RGD induced a pronounced reduction in cell viability (Figure 9). In contrast, the presence of FAAs markedly reduced cytotoxic effects. In particular, the trifluorinated conjugate maintained high cell viability even at concentrations that support supramolecular assembly, indicating that fluorination mitigates adverse cellular responses associated with peptide aggregation. Further increases in fluorine content led to a modest decrease in viability; however, all fluorinated conjugates remained above established cytotoxicity thresholds.

**FIGURE 9:**
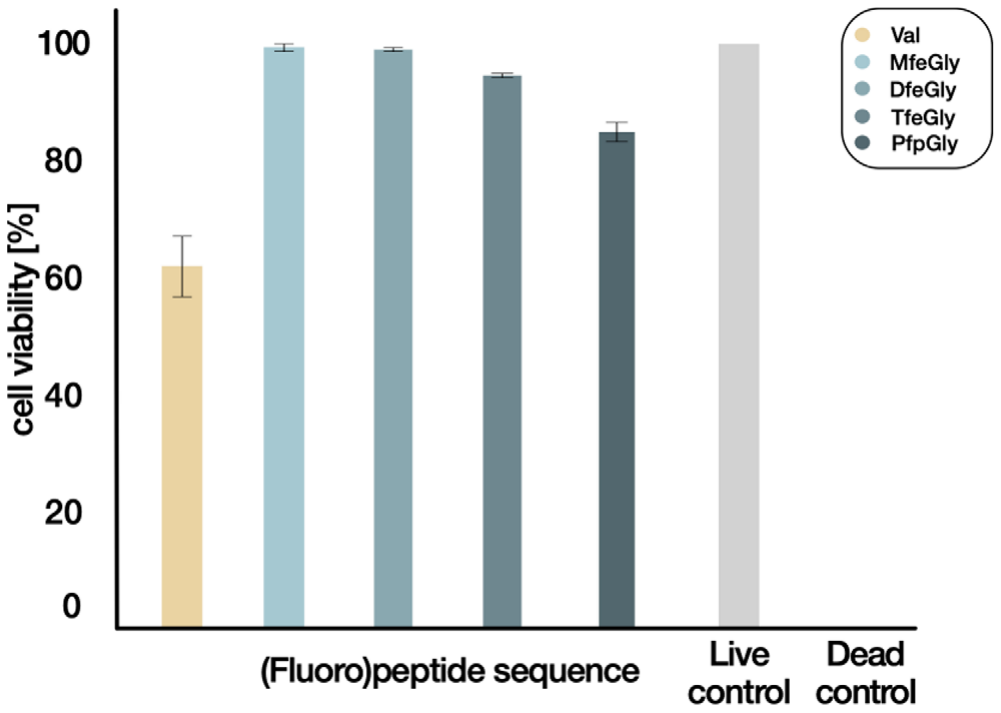
Cell viability (%) of A549 cells treated with amphiphilic oligopeptides at the operative concentration (0.1 wt.%), showing the effect of increasing fluorination degree. Data are reported as Mean ± SE, *n* = 6.

At this concentration, the peptide conjugates adopt distinct supramolecular architectures, allowing to directly assess structure–property relationships under biologically relevant conditions. Herein, cytotoxicity strongly correlates with the type of supramolecular assembly formed. Among the systems investigated, only the non‐fluorinated control Val6RGD forming amyloid‐like fibrils exhibited pronounced toxicity, whereas fluoropeptides assembling into more dynamic, hydrated nanostructures (TfeGly6RGD) showed negligible effects on cell viability. This observation suggests that cytotoxicity is not an intrinsic property of the peptide sequence itself, but rather emerges from the specific supramolecular organization. This distinction is particularly relevant for nanocarrier design, where avoidance of amyloid‐like packing is critical for biocompatibility [24].

Amyloid‐like fibrillar structures are known to interact with and disrupt cellular membranes, either through direct membrane insertion or via the formation of intermediate oligomeric species. In contrast, structurally dynamic assemblies are less likely to form such membrane‐active species, which may account for their improved biocompatibility. This behavior is consistent with previous reports on aggregating bioactive peptides, where β‐sheet‐rich, amyloid‐like assemblies are frequently associated with cytotoxic effects, while more hydrated and less ordered nanostructures tend to exhibit reduced biological impact [25].

These results demonstrate that fluorination systematically improves cellular compatibility across the conjugate series, while identifying an optimal fluorination regime that combines robust assembly with minimal cytotoxicity. Notably, this optimum corresponds to the trifluorinated conjugate TfeGly6RGD, consistent with trends observed across structural, mechanistic, and functional analyses throughout this study. In combination with their enzymatic degradability, the fluorinated assemblies thus fulfill key criteria for biocompatible, peptide‐based carrier systems. Having established the biocompatibility of the fluoropeptide assemblies and identified an optimal fluorination regime, we next investigated whether these structural features translate into effective drug encapsulation, controlled release, and receptor‐specific cellular uptake.

### 2.9 In Vitro Drug Release and Receptor‐Specific Cellular Uptake

The pH‐responsive SA of the amphiphilic peptide conjugates suggests their suitability as carriers for intracellular drug delivery. Given the acidic microenvironment of endosomes and tumor tissues, we selected doxorubicin (DOX) as a model cargo to evaluate encapsulation efficiency, pH‐triggered release, and cellular uptake [26]. Based on the carrier screening and biocompatibility assessment, the non‐fluorinated reference Val6RGD and the optimized trifluorinated conjugate TfeGly6RGD were investigated as representative systems.

The pH‐dependent release of DOX was first validated via a dialysis‐based in vitro assay to confirm that the trends observed with pyrene translate to a therapeutically relevant cargo. At pH 7.4, peptide assemblies formed at 0.1 wt.% retained the drug efficiently, consistent with the structural stability of the assembled state under neutral medium conditions. Upon shifting to acidic medium pH, a rapid and pronounced DOX release was observed, consistent with protonation‐induced electrostatic repulsion and disassembly of the peptide assemblies (Figure 10A). While the release kinetics were comparable across the variants, TfeGly6RGD exhibited a substantially higher drug loading capacity, corresponding to an approximately 94% increase, in agreement with the enhanced encapsulation observed for pyrene, suggesting that fluorination promotes more efficient cargo partitioning within the assemblies without significantly altering release dynamics (Figure 10B).

**FIGURE 10:**
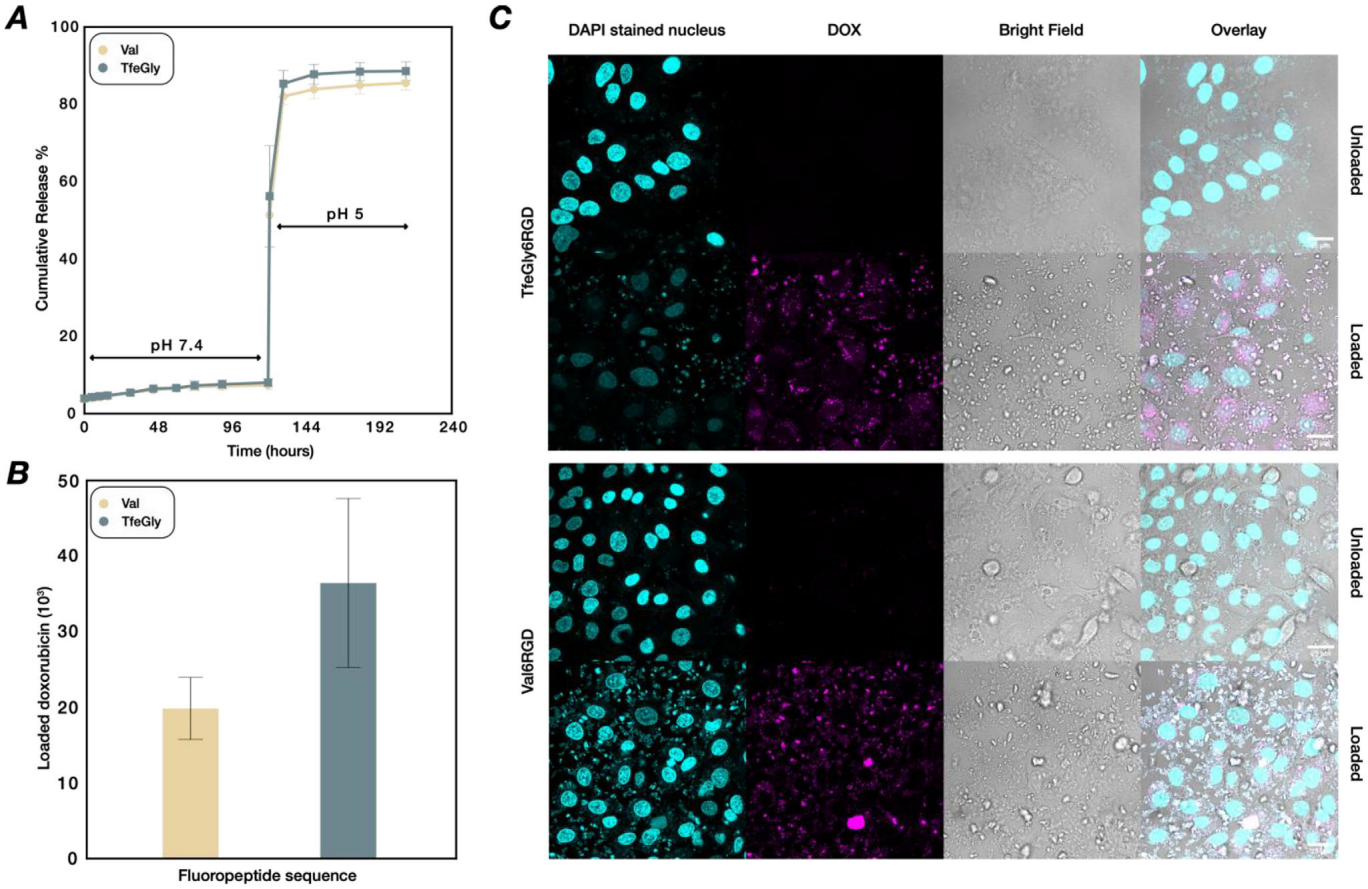
(A) DOX release behavior of (fluorinated)oligopeptide self‐assembled fibrils in neutral medium (pH 7.4) and acidic medium (pH 5). (B) Total doxorubicin loading in Val6RGD and TfeGly6RGD, calculated as the sum of cumulative release and the amount of drug still encapsulated at the end of the experiment. (C) CSLM‐based cellular uptake assay for TfeGly6RGD (top) and Val6RGD (bottom). Data are reported as Mean ± SE, *n* = 3.

Cellular uptake was evaluated in A549 cells, which express integrins targeted by the RGD motif, following incubation with DOX‐loaded peptides at 0.1 wt.% for 20 h. Confocal laser scanning microscopy (CLSM) revealed efficient intracellular DOX fluorescence predominantly localized in the perinuclear region (Figure 10C). Given the presence of an RGD‐derived motif and the expression of integrins in A459 cells, receptor‐mediated uptake represents a plausible mechanism. However, the involvement of specific integrin‐mediated endocytosis pathways was not directly investigated in this study. While nuclear accumulation of DOX is limited, the observed perinuclear localization is consistent with intracellular trafficking through endosomal compartments, as commonly reported for nanocarrier‐based delivery systems.

Notably, differences in supramolecular structure are also reflected in drug release outcomes. The trifluorinated conjugate, which forms more dynamic and hydrated assemblies, shows efficient uptake and DOX delivery, whereas the amyloid‐like fibril‐forming peptide Val6RGD exhibits reduced suitability as a carrier, consistent with its rigid packing and previously observed cytotoxicity. In addition, reduced DAPI staining intensity in cells treated with DOX‐loaded TfeGly6RGD peptides compared to controls indicates compromised cell viability, supporting successful intracellular delivery of the cytotoxic payload. However, quantitative evaluation of cellular uptake is not feasible in these conditions, as the amyloid‐forming non‐fluorinated control Val6RGD exhibited enhanced cytotoxicity at the applied concentration.

Taken together, these results demonstrate that the performance differences observed among the fluorinated analogues arise from systematic fluorine‐dependent modulation of intermolecular interactions governing SA. In particular, the trifluorinated conjugate (TfeGly6RGD) establishes an optimal balance between hydrophobic and hydrophilic interactions, enabling stable yet dynamically hydrated fibrillar assemblies. This intermediate regime suppresses β‐sheet and amyloid packing while maintaining sufficient structural integrity for efficient cargo encapsulation. In contrast, the non‐fluorinated analogue exhibits more rigid amyloid assemblies, resulting in inferior performance in drug loading, release behavior, and cellular compatibility. The enhanced hydration and dynamic packing observed for TfeGly6RGD correlate with improved biocompatibility and more effective pH‐responsive drug release. Collectively, these findings identify fluorination as a tunable molecular parameter that governs the interplay between assembly stability and structural dynamics, thereby determining nanocarrier performance.

## 3 Conclusion

In this work, we establish fluorination as a molecular design parameter for programming the SA, biocompatibility, and functional performance of peptide‐based nanocarriers. By systematically varying the fluorine content within an amphiphilic peptide–RGD conjugate architecture, we demonstrate that fluorination reshapes peptide interactions across multiple length scales, spanning secondary structure formation, supramolecular morphology, and biological response. Structural and mechanistic analyses reveal that fluorination redirects peptide assembly away from rigid, amyloid‐like fibrils toward compact yet hydrated supramolecular architectures. Carrier studies identify the formation of core–shell assemblies as a prerequisite for hydrophobic cargo encapsulation, while MD simulations provide a mechanistic rationale for how fluorine‐specific interactions decouple backbone association from cooperative β‐sheet formation. Structural design features are reflected in biological performance. Fluorination systematically improves cellular compatibility and preserves enzymatic degradability, highlighting the transient and biodegradable nature of the assemblies. The amyloid‐like, non‐fluorinated control system exhibited strong cytotoxicity, thereby making it unsuitable for effective cargo applications. However, among the investigated series, an optimal fluorination regime emerges at the trifluorinated conjugate, which consistently combines robust dynamic self‐assembly, minimal cytotoxicity, efficient drug loading, and pH‐responsive release, while cellular imaging indicates intracellular delivery in A549 cells consistent with RGD‐assisted uptake. Together, these findings demonstrate that fluoropeptides constitute a versatile and tunable platform for the rational design of peptide‐based nanocarriers. More broadly, they illustrate that fluoropeptides provide an orthogonal functionality in peptide design, which allows precise control over side‐chain chemistry that can be leveraged to integrate structural programmability with functional delivery performance, opening new opportunities for the development of biodegradable, targeted drug delivery systems. The fluorination‐guided design principles established in this work are expected to be broadly applicable beyond the specific RGD‐functionalized peptide amphiphiles investigated here. In particular, fluorine‐mediated tuning of hydrophobic–hydrophilic balance and intermolecular packing may be extended to other classes of self‐assembling peptides, including β‐sheet‐forming peptide hydrogels, nanofibers, and modular peptide systems for a wide variety of biomedical applications. By enabling precise control over assembly hydration and structural dynamics, fluorination offers a generalizable strategy to modulate loading capacity, stability, and biological interactions across diverse peptide‐based nanomaterials. Moreover, this approach may be leveraged for the design of next‐generation delivery systems for a wide range of therapeutic cargos, including small molecules, peptides, and nucleic acids, thereby expanding the utility of fluoropeptide architectures in targeted and stimuli‐responsive biomedical applications.

## Conflicts of Interest

The authors declare no conflicts of interest.

## Supporting information




**Supporting File**: smll74993‐sup‐0001‐SuppMat.docx.

## Data Availability

The data that support the findings of this study are available from the corresponding author upon reasonable request.
