# Supplementary material for: Fluorination as a Molecular Design Parameter for Programming Peptide Nanocarriers
Source: Small. 2026 Aug 2;22(56):e74993. doi: 10.1002/smll.74993 (PMC13653353; doi:10.1002/smll.74993)
Supplement: Supplementary file 1 [file SMLL-22-e74993-s001.docx]

**Fluorination as a Molecular Design Parameter for Programming Peptide Nanocarriers**

Maurizio Iannuzzi^[a]^, Holly Fiedler^[a]^, Kilian Haoues^[a]^, Suvrat Chowdhary^[a]^, Andreas F. Thünemann^[b]^, Boris Schade^[c]^, Hossein Batebi^[d]^, Elisa Quaas^[e]^, Roland R. Netz^[d]^ and Beate Koksch*^[a]^

**SUPPORTING INFORMATION**

TABLE OF CONTENTS

[Gram-scale synthesis and characterization of fluorinated amino acids	2](#_Toc228276305)

[Synthesis of Ni(II) complex (4)	4](#_Toc228276306)

[Synthesis of CF3CH2-Ni(II) complex (5)	5](#_Toc228276307)

[Synthesis of Fmoc-TfeGly-OH (6)	7](#_Toc228276308)

[Synthesis of CF3CF2CH2-Ni(II) complex (11)	9](#_Toc228276309)

[Synthesis of Fmoc-PfpGly-OH (12)	11](#_Toc228276310)

[Synthesis of CFH2CH2-Ni(II) complex (7)	13](#_Toc228276311)

[Synthesis of Fmoc-MfeGly-OH (8)	14](#_Toc228276312)

[Synthesis of CF2HCH2-Ni(II) complex (9)	15](#_Toc228276313)

[Synthesis of Fmoc-DfeGly-OH (10)	16](#_Toc228276314)

[Peptide synthesis and purification methods	17](#_Toc228276315)

[Val4RGD	19](#_Toc228276316)

[Val6RGD	20](#_Toc228276317)

[Val8RGD	21](#_Toc228276318)

[MfeGly6RGD	22](#_Toc228276319)

[DfeGly6RGD	23](#_Toc228276320)

[TfeGly4RGD	24](#_Toc228276321)

[TfeGly6RGD	25](#_Toc228276322)

[TfeGly8RGD	26](#_Toc228276323)

[PfpGly4RGD	27](#_Toc228276324)

[PfpGly6RGD	28](#_Toc228276325)

[RP-HPLC assay for estimation of hydrophobicity	29](#_Toc228276326)

[Peptide stock solution, concentration determination and sample preparation	29](#_Toc228276327)

[Extensive CD analysis	30](#_Toc228276328)

[Further Cryo-TEM images	32](#_Toc228276329)

[SAXS data analysis	35](#_Toc228276330)

[Thioflavin T (ThT) fluorescence assay for amyloid detection	38](#_Toc228276331)

[Extensive MD analysis	39](#_Toc228276332)

[Peptide digestion assay (Real time monitoring)	45](#_Toc228276333)

[Carrier properties: pyrene encapsulation experiments	47](#_Toc228276334)

[Doxorubicin drug loading in vitro release monitoring method	48](#_Toc228276335)

[Cell experiments	49](#_Toc228276336)

[Cytotoxicity test - live/dead assay	49](#_Toc228276337)

[Intracellular uptake	52](#_Toc228276338)

# **Gram-scale synthesis and characterization of fluorinated amino acids**

Incorporating fluorine into organic compounds, such as aliphatic amino acids, has been of great interest due to fluorine’s unique behavior in modifying their properties. In the last 40 years, a novel approach has been developed using chiral nickel (II) complexes of glycine or alanine – Schiff bases to access non canonical amino acids. The Soloshonok trichlorinated complex **4** displayed great potential in synthesizing fluorinated tailor-made amino acids, drastically improving the synthetic accessibility to these crucial building blocks enhancing the stereoselectivity of the alkylation step^27^.

In this context, a gram-scale synthesis of fluorenylmethoxycarbonyl (Fmoc) protected trigluoroethylglycine (TfeGly) was demonstrated by Soloshonok and co-workers^28^.

**Scheme 1**: Synthesis of Fmoc-TfeGly-OH according to Soloshonok et al.

In the first step, H-Pro-OH (**1**) undergoes selective N-alkylation with 3,4-dichlorobenzyl chloride in a basic KOH/isopropanol solution, ensuring that the carboxylic acid group remains unreacted. The resulting intermediate (**2**) is then converted to the corresponding acid chloride using PCl₅, which allows for amide bond formation with 2-amino-5-chlorobenzophenone, yielding compound **3**. Compound 3 is then reacted with H-Gly-OH, nickel(II) acetate tetrahydrate, and DBU to form the Schiff base ligand complex (**4**). Asymmetric alkylation of the chiral Ni-complex **4** with CF₃CH₂I, followed by complex disassembly and simultaneous Fmoc protection, leads the final product: the Fmoc-protected (2S)-4,4,4-trifluoroethylglycine (Fmoc-TfeGly-OH, **6**).

In the past years we extended the synthetic approach proposed by Soloshonok et al. in order to access a variety of linear, β- and γ-branched and aromatic amino acids.^15,16^

**Figure S1**: Chemical structures of the fluorinated amino acids synthesized and characterized in the context of this work.

The fluorinated amino acids (2S)-4-monofluoroethylglycine (MfeGly), (2S)-4,4-difluoroethylglycine (DfeGly), (2S)-4,4,4-trifluoroethylglycine (TfeGly) and (2S)-5,5,5,4,4-pentafluoropropylylglycine (PfpGly) were synthesized according to the published procedures.

**Scheme 2**: Optimized conditions according to Hohmann et al. for the synthesis of Fmoc-protected fluorinated amino acids MfeGly, DfeGly, TfeGly and PfpGly.

## **Synthesis of Ni(II) complex (4)**

NI(II) complex was synthetized according to Soloshonok et al. (Scheme 1) and its purity characterized via NMR and mass spectrometry.

**^1^H NMR (600 MHz, MeOH-d_4_)** δ 8.79 (d, *J* = 2.1 Hz, 1H), 8.31 (dd, *J* = 8.2, 2.1 Hz, 1H), 8.04 – 7.95 (m, 1H), 7.64 (t, *J* = 7.2 Hz, 1H), 7.60 – 7.51 (m, 3H), 7.28 (d, *J* = 7.3 Hz, 1H), 7.14 (dd, *J* = 9.2, 2.6 Hz, 1H), 7.04 (d, *J* = 6.8 Hz, 1H), 6.69 (d, *J* = 2.6 Hz, 1H), 4.23 (d, *J* = 12.5 Hz, 1H), 3.64 (s, 1H), 3.54 (dd, *J* = 11.1, 5.8 Hz, 1H), 3.51 – 3.37 (m, 3H), 2.63 – 2.52 (m, 1H), 2.51 – 2.42 (m, 1H), 2.23 – 2.12 (m, 2H).

## **Synthesis of CF_3_CH_2_-Ni(II) complex (5)**

The chiral Ni (II) complex **4** (30.0 g, 49.9 mmol, 1.00 equiv.) and 1,1,1-trifluoro-2-iodoethane (5.20 mL, 52.4 mmol, 1.05 equiv.) were suspended in deoxygenated, dry DMF (300 mL) under N_2_ atmosphere. After cooling the solution to 0 ^o^C, KOH (3.00 g, 52.4 mmol, 1.05 equiv.) dissolved in deoxygenated MeOH (28 mL) was added. The solution was stirred for 3.5 h at 23 ^o^C. The red solution was quenched with H_2_O (110 mL) and stirred for 1 h. H_2_O (40 mL) was added, and the solution was further stirred for 16 h. The orange precipitate was filtered and washed with a DMF:H2O mixture (2:1, 60 mL) and H2O (80 mL). Finally, the received product was dried in vacuo at 55 ^o^C. The alkylated Ni (II) complex **5** was obtained as a red solid without further purification (24.56 g, 36.0 mmol, 73%).

**1H NMR (600 MHz, MeOH-d4):** δ = 8.82 (d, J = 2.1 Hz, 1H), 8.28 (dd, J = 8.2, 2.1 Hz, 1H), 8.00 (d, J = 9.3 Hz, 1H), 7.70 – 7.61 (m, 2H), 7.57 (td, J = 7.5, 1.5 Hz, 1H), 7.51 (dt, J = 7.4, 1.7 Hz, 1H), 7.47 (d, J = 8.2 Hz, 1H), 7.12 (dd, J = 9.3, 2.6 Hz, 1H), 7.00 (dt, J = 7.8, 1.6 Hz, 1H), 6.53 (d, J = 2.6 Hz, 1H), 4.26 (dd, J = 7.1, 3.1 Hz, 1H), 4.19 (d, J = 12.6 Hz, 1H), 3.57 (dd, J = 10.7, 6.5 Hz, 1H), 3.49 – 3.34 (m, 3H), 2.61 – 2.50 (m, 1H), 2.35 – 2.25 (m, 1H), 2.23 – 2.09 (m, 2H) ppm.

**^19^F NMR** (565 MHz, MeOH-d4): δ = -61.60 (t, J = 10.4 Hz) ppm.

## **Synthesis of Fmoc-TfeGly-OH (6)**

The alkylated Ni (II) complex **5** (24.56 g, 36.0 mmol, 1.00 equiv.) was dissolved in DME (140 mL) and an aqueous 3M HCl solution (85 mL). The reaction mixture was stirred for 2 h at 60 ^o^C. Afterward, the green mixture was filtered and washed with water (50 mL). Subsequently, the solution was concentrated and further chlorinated BPB ligand **3** precipitated. The mixture was filtered and washed with water (25 mL). The recyclable chlorinated BPD ligand **3** was isolated as a white solid (15.17 g, 31.1 mmol, 86%). Acetonitrile (MeCN) (85 mL) and ethylenediaminetetraacetic acid (EDTA)-(Na_2_) (13.40 g, 36.0 mmol, 1.00 equiv.) were added to the solution. The solution was stirred for 2 h at 23 ^o^C. Afterward, the pH of the solution was adjusted to 8 using an aqueous 48%-NaOH solution. Subsequently, after addition of Na_2_CO_3_ (7.63 g, 72.0 mmol, 2.00 equiv.), Fmoc-OSu (12.14 g, 36.0 mmol, 1 equiv.) dissolved in tetrahydrofuran (THF) (70 mL) was added dropwise over 30 min. The resulting mixture was stirred at 23 ^o^C for 20 h. Under reduced pressure, MeCN and THF were removed from the solution. After the precipitation of a white solid, H_2_O (1000 mL) was added to redissolve the precipitate. The obtained solution was washed with diethyl ether (Et_2_O) (4 x 50 mL). The pH of the aqueous solution was adjusted to 2 with 37% - aqueous HCl and additionally extracted

with EtOAc (3 x 150 mL). The organic phases were dried over Na_2_SO_4_, filtered, concentrated, and dried under reduced pressure. The crude product was recrystallized from EtOAc and hexane, filtered, and washed with ice-cold hexane (80 mL). Finally, the received product was dried in vacuo at 40 ^o^C. Fmoc-TfeGly **6** was isolated as a white solid (8.28 g, 27.0 mmol, 75%).

**^1^H NMR (600 MHz, MeOH-d4): δ** = 7.76 (d, J = 7.6 Hz, 2H), 7.68 – 7.59 (m, 2H), 7.36 (t, J = 7.5 Hz, 2H), 7.27 (t, J = 7.5 Hz, 2H), 4.46 (dd, J = 10.0, 3.4 Hz, 1H), 4.31 (d, J = 7.2 Hz, 2H), 4.20 (t, J = 7.1 Hz, 1H), 2.87 – 2.77 (m, 1H), 2.69 – 2.55 (m, 1H) ppm.

**^19^F NMR (565 MHz, MeOH-d4):** δ = -65.79 (t, J = 10.6 Hz) ppm.

## **Synthesis of CF_3_CF_2_CH_2_-Ni(II) complex (11)**

The chiral Ni (II) complex 30 (22.1 g, 36.7 mmol, 1.00 equiv.) was suspended in dry, deoxygenated DMF (90 mL) under N_2_ atmosphere. The mixture was cooled to 0^o^C and 1,1,1,2,2-pentafluoro-3-iodopropane (10.0 g, 38.5 mmol, 1.05 equiv.) and NaH (60% dispersion in mineral oil, 1.91 g, 47.7 mmol, 1.30 equiv.) were added to the suspension. The reaction mixture was stirred for 2 h at 0 ^o^C. The red solution was quenched with H_2_O (41 mL) and stirred for 1 h. Additionally, H_2_O (19 mL) was added, and the solution was further stirred for 16 h. The red precipitate was filtered and washed with a DMF: H_2_O mixture (2:1, 22 mL) and H_2_O (30 mL). The received compound was dried in vacuo at 55^o^C. The crude product was recrystallized from EtOH. Finally, the received product was dried in vacuo at 55 ^o^C. The alkylated Ni (II) complex **11** was obtained as a red solid without further purification (11.15 g, 15.0 mmol, 41%).

­­

**^1^H NMR (600 MHz, CHCl_3_-d):** δ = 8.94 (s, 1H), 8.16 (d, J = 9.4 Hz, 1H), 7.72 (d, J = 8.1 Hz, 1H), 7.65 – 7.54 (m, 2H), 7.51 (d, J = 7.6 Hz, 1H), 7.36 (d, J = 8.1 Hz, 1H), 7.30 (d, J = 6.3 Hz, 1H), 7.13 (d, J = 9.3 Hz, 1H), 6.88 (d, J = 7.6 Hz, 1H), 6.57 (s, 1H), 4.28 (dd, J = 16.7, 9.7 Hz, 2H), 3.58 (t, J = 8.6 Hz, 1H), 3.44 (q, J = 10.2, 9.6 Hz, 1H), 3.34 (t, J = 8.4 Hz, 1H), 3.23 (d, J = 12.7 Hz, 1H), 2.76 (t, J = 10.1 Hz, 1H), 2.65 – 2.36 (m, 2H), 2.16 (d, J = 10.5 Hz, 1H), 2.11 – 1.89 (m, 2H) ppm.

**^19^F NMR (565 MHz, CHCl_3_-d):** δ = -86.07, -112.54 (dd, J = 261.0, 34.7 Hz), -115.35 (dd, J = 260.7, 32.8 Hz) ppm.

## **Synthesis of Fmoc-PfpGly-OH (12)**

The alkylated Ni (II) complex 34 (11.15 g, 15.0 mmol, 1.00 equiv.) was dissolved in DME (53 mL) and an aqueous 3M HCl solution (25 mL). The reaction mixture was stirred for 3 h at 60 ^o^C. Afterward, the green mixture was filtered and washed with water (20 mL). Subsequently, the solution was concentrated and further chlorinated BPD ligand **3** precipitated. The mixture was filtered and washed with water (19 mL). The recyclable chlorinated BPD ligand **3** was isolated as a white solid (6.00 g, 12.3 mmol, 82%). MeCN (34 mL) and EDTA-(Na2) (5.58 g, 15.0 mmol, 1.00 equiv.) were added to the solution. The reaction mixture was stirred for 2 h at 23 ^o^C. Subsequently, the pH of the solution was adjusted to 7 using an aqueous 48%-NaOH solution. A white solid precipitated; therefore, a MeCN:H_2_O mixture (1:1, 200 mL) was added to redissolve the solid. Subsequently, Na_2_CO_3_ (3.20 g, 30.0 mmol, 2.00 equiv.) was added. H_2_O (100 mL) was added to dissolve the precipitated solid further. Fmoc-OSu (5.10 g, 15.0 mmol, 1 equiv.) dissolved in acetone (60 mL) was added dropwise over 30 min. The resulting mixture was stirred at 23^o^C for 20 h. Under reduced pressure, MeCN and acetone were removed from the solution. After the precipitation of a white solid, H_2_O (400 mL) was added to redissolve the precipitate. The obtained solution was washed with Et_2_O (3 x 50 mL). The pH of the aqueous solution was adjusted to 2 with 37%-aqueous HCl and additionally extracted with EtOAc (3 x 150 mL). The organic phases were dried over Na_2_SO_4_, filtered, and concentrated. After washing with Et2O, a TLC of the collected organic phases showed the presence of the crude product. Therefore, the Et_2_O and the EtOAc phases were combined, concentrated, and dried under reduced pressure. The crude product was purified via flash column chromatography (EtOAc:hexane (containing 1% (v/v) AcOH) = 10:1 - 6:1 - 4:1 - 2:1). Finally, the received product was concentrated and dried in vacuo at 50 ^o^C. Fmoc-PfpGly 32 was isolated as a white solid (1.56 g, 3.6 mmol, 24%).

**1H NMR** (600 MHz, MeOH-d4): δ = 7.78 (d, J = 7.6 Hz, 1H), 7.66 (d, J = 8.5 Hz, 1H), 7.38 (t, J = 7.5 Hz, 1H), 7.29 (t, J = 7.5 Hz, 1H), 4.60 – 4.54 (m, 1H), 4.35 (d, J = 7.2 Hz, 1H), 4.22 (t, J = 7.1 Hz, 1H), 2.87 – 2.74 (m, 0H), 2.71 – 2.58 (m, 1H) ppm.

**^19^F NMR** (565 MHz, MeOH-d4): δ = -85.92, -117.00 (ddd, J = 267.3, 28.2, 9.5 Hz), -118.05 (ddd, J = 267.3, 26.8, 9.3 Hz) ppm.

## **Synthesis of CFH_2_CH_2_-Ni(II) complex (7)**

Under inert conditions, a 500 mL round flask was charged with Ni(II) complex **4** (20.0 g, 33.2 mmol, 1.00 equiv.), alkyl iodide **7** (6.06 g, 34.86 mmol, 1.05 equiv.) and dry, degassed DMF (200 mL). At 0 °C, potassium hydroxide (2.00 g, 34.86 mmol, 1.05 equiv.) in dry, degassed methanol (18.0 mL) was added, and the reaction mixture was stirred for 2 h, while warming up to 23 °C. Subsequently, the reaction was quenched by addition of H_2_O (80 mL) and was stirred for 1 h. Then, another amount of H_2_O (60 mL) was added and the reaction mixture was left to stir for 1 h. The precipitated solid was filtered off, washed with H_2_O:DMF (1:2, 100 mL) and dried *in vacuo* at 55 °C. The product was obtained as a red solid (18.58 g, 86%).

**^19^F NMR (565 MHz, CD_3_OD):** δ -218.42 (tdd, *J* = 46.7, 31.7, 22.2 Hz) ppm.

## **Synthesis of Fmoc-MfeGly-OH (8)**

A 500 mL round flask was charged with alkylated complex 9 (18.50 g, 28.6 mmol, 1.00 equiv.) and DME (95.0 mL). At 60 °C, 3M HCl solution (50 mL) was added and the reaction mixture was stirred for 2 h. The precipitated BPD ligand **3** was filtered off and washed with water (50 mL) and the filtrates were concentrated under reduced pressure. This led to more precipitation of BPD ligand **3**, which was then filtered off. To the combined filtrates, EDTA‑Na_2_ (9.67 g, 28.6 mmol, 1.00 equiv.) in acetonitrile (57 mL), was added and the reaction mixture was stirred at 23 °C for 2 h. After completion, the reaction was quenched by adjusting the pH of the reaction mixture to 8 by usage of 48%‑NaOH solution. Subsequently, sodium carbonate (6.06 g, 57.2 mmol, 2.00 equiv.) was supplied, FmocOSu (9.65 g, 28.6 mmol, 1.00 equiv.) in acetone (114 mL) was added dropwise and the reaction mixture was stirred at 23 °C for 20 h. Afterward, acetonitrile and acetone were removed from the reaction mixture under reduced pressure. The aqueous solution was washed with Et_2_O (3 x 100 mL) to remove any leftover FmocOSu. Then, the pH of the solution was adjusted to 2 by using 2M HCl solution. Next, the mixture was extracted with ethyl acetate (6 x 80 mL) and the combined organic phases were dried over Na_2_SO_4_. After filtration, the crude product was dried in vacuo at 55 °C. The crude product was purified via recrystallization from ethyl acetate and obtained as a white powder (5.52 g, 56%).

**^19^F NMR (565 MHz, Me_3_OH-d_4_):** δ -222.45 – -222.80 (m) ppm.

## **Synthesis of CF_2_HCH_2_-Ni(II) complex (9)**

Under inert conditions, a 500 mL round flask was charged with Ni(II) complex **4** (20.0 g, 33.2 mmol, 1.00 equiv.), alkyl iodide **8** (6.70 g, 34.90 mmol, 1.05 equiv.) and dry, degassed DMF (200 mL). At 0 °C, potassium hydroxide (2.00 g, 34.90 mmol, 1.05 equiv.) in dry, degassed methanol (18.0 mL) was added, and the reaction mixture was stirred for 2 h, while warming up to 23 °C. Subsequently, the reaction was quenched by addition of H_2_O (80 mL) and was stirred for 1 h. Then, another amount of H_2_O (60 mL) was added and the reaction mixture was left to stir for 1 h. The precipitated solid was filtered off, washed with H_2_O/DMF (1:2, 100 mL) and dried *in vacuo* at 55 °C. The product was obtained as a red solid (19.13 g, 86%).

**^19^F NMR (565 MHz, CHCl_3_-d):** δ -113.97 (dddd, *J* = 287.8, 55.9, 15.4, 10.0 Hz), -115.95 (dddd, *J* = 287.7, 55.6, 23.9, 14.0 Hz) ppm.

## **Synthesis of Fmoc-DfeGly-OH (10)**

A 500 mL round flask was charged with alkylated complex **10** (19.10 g, 28.7 mmol, 1.00 equiv.) and DME (96.0 mL). At 60 °C, 3M HCl solution (50 mL) was added and the reaction mixture was stirred for 2 h. The precipitated BPD ligand **3** was filtered off and washed with water (50 mL) and the filtrates were concentrated under reduced pressure. This led to more precipitation of BPD ligand **3**, which was then filtered off. To the combined filtrates, EDTA‑Na_2_ (9.71 g, 28.7 mmol, 1.00 equiv.) in acetonitrile (57 mL), was added and the reaction mixture was stirred at 23 °C for 2 h. After completion, the reaction was quenched by adjusting the pH of the reaction mixture to **8** by usage of 48%‑NaOH solution. Subsequently, sodium carbonate (6.08 g, 57.4 mmol, 2.00 equiv.) was supplied, FmocOSu (9.68 g, 28.7 mmol, 1.00 equiv.) in acetone (114 mL) was added dropwise and the reaction mixture was stirred at 23 °C for 20 h. Afterward, acetonitrile and acetone were removed from the reaction mixture under reduced pressure. The aqueous solution was washed with Et_2_O (3 x 100 mL) to remove any leftover FmocOSu. Then, the pH of the solution was adjusted to 2 by using 2M HCl solution. Next, the mixture was extracted with ethyl acetate (6 x 80 mL) and the combined organic phases were dried over Na_2_SO_4_. After filtration, the crude product was dried in vacuo at 55 °C. The crude product was purified via recrystallization from ethyl acetate/n‑hexane and obtained as a white powder (2.00 g, 20%).

**^19^F NMR (565 MHz, MeOH-d_4_)**: δ -117.44 – -118.98 (m) ppm.

# **Peptide synthesis and purification methods**

All peptides were synthesized with a microwave-equipped Liberty Blue^TM^ peptide synthesizer (CEM, Matthews, NC, USA) on a 0.1 or 0.05 mmol scale using a preloaded Fmoc-serine(tBu) Wang resin. Coupling steps were performed using oxyma/DIC as activating reagents (0.05 mmol scale: 0.5 M oxyma in DMF and 0.25 M DIC in DMF/0.1 mmol scale: 1 M oxyma in DMF and 0.5 M DIC in DMF). Coupling of native Fmoc-protected amino acids occurred in DMF using 5 eq. of substance with 5 eq. of activating reagents and double couplings of 4 min coupling time. Non-canonical amino acids, MfeGly, DfeGly, TfeGly and PfpGly were introduced with a tailor-made coupling cycle (**Table ST1**) reported by Leppkes *et al^29^* then adapted for the synthesis of highly fluorinated peptides by Chowdhary^20^ and Hohmann^21^ which uses prolonged microwave heating time, reduced equivalents of the amino acid (1.5 eq.) employed and additional washing steps between Fmoc deprotection and coupling step. To enhance the performance and the consequent final yield 3 eq. of FAAs were used in this case. For deprotection of the N-terminus, a 10% piperazine (w/v) solution in EtOH/NMP (1:9) was used. All peptides were cleaved from the resin by treatment with TFA/TIPS/H_2_O (95/2.5/2.5) for three hours shaking at room temperature. Then the resins were washed with TFA and DCM, and excess of solvents were removed by evaporation. Peptides were dried by lyophilization before purification with preparative reversed phase HPLC. Purification of synthesized peptides was performed on a Nexera Prep from Shimadzu (Kyoto, Japan). It comprised a system controller SCL-40, a UV-vis detector SPD-40 with a flow cell 40UV of 10 mm pathlength, a preparative liquid chromatograph LC-20AP including a mixing chamber, a prep quaternary valve FCV-200AL, and a liquid handler LH-40. A Kinetex RPC18 end capped (5 μM, 100 Å, 250 × 21.2 mm, Phenomenex®, USA) HPLC-column was used. A Security GuardTM PREP Cartridge Holder Kit (21.20 mm, ID, Phenomenex®, USA) served as pre-column. As eluents water and ACN, both containing 0.1% (v/v) TFA were applied. HPLC runs were performed with a flow rate of 15.0 mL min^-1^, UV- detection occurred at 220 nm for respective peptides. Data analysis occurred with an EZChrom Elite Software (Version 3.3.2 SP2, Agilent). After separation, the purity of the collected fractions was determined by analytical HPLC. Analytical HPLC was carried out on a Chromaster 600 bar DAD-System with CSM software or a Hitachi PrimaideTM UV-HPLC system (both from VWR/Hitachi, Darmstadt, Germany). A Kinetex® RP-C18 (5 μM, 100 Å, 250 × 4.6 mm, Phenomenex®, USA) column and a SecurityGuardTM Cartridge Kit equipped with a C18 cartridge (4 × 3.0 mm, Phenomenex®, USA) as pre-column was used. As eluents water and ACN, both containing 0.1% (v/v) TFA were applied. A flow rate of 1 mL min^-1^ was used and UV-detection occurred at 220 nm or 280 nm for respective peptides. Data analysis was done with EZ Chrom ELITE software (version 3.3.2, Agilent). The resulting pure peptides (>98%) were obtained after lyophilization of the collected fractions.

**Table ST1:** Special cycle for automated microwave-assisted SPPS with 0.1 mmol scale on Liberty Blue peptide synthesizer for the coupling of fluorinated amino acids. All reagents were dissolved in DMF.

|  | **Reagents** | | | **Conditions** | | |
| --- | --- | --- | --- | --- | --- | --- |
| **Step** |  | | |  | | |
|  | **Name** | **Conc. [M]** | **Volume [mL]** | **T [^o^C]** | **Power [W]** | **Time [s]** |
| **Deprotection*** | Piperazine  Dipea | 10 wt%  0.1 | 4 | 75  90 | 155  30 | 15  60 |
| **Washing (9 cycles)** | DMF | - | 4 | 25 | - | 5 |
| **FAA coupling** | Fmoc-F(AA)  DIC  Oxyma  DIPEA | 0.12  1  1  0.1 | 2.5  1  0.5 | 75  90 | 217  43 | 15  585 |
| **Washing** | DMF | - | 4 | 25 | - | 5 |
| *EtOH/NMP, 1/9 v/v | | | | | | |

**Table ST2**: Sequences of fluoropeptide conjugates involved in the screening phase, yields and reference masses

| **Peptide** | **Sequence** | **Yield (%)** | **Reference mass (Da)** |
| --- | --- | --- | --- |
| Val4RGD | NH2-(Val)_4_GYGKKGRGDS-OH | 54 | 1420.78 |
| Val6RGD | NH2-(Val)_6_GYGKKGRGDS-OH | 48 | 1618.90 |
| Val8RGD | NH2-(Val)_8_ GYGKKGRGDS-OH | Traces | 1817.17 |
| MfeGly6RGD | NH2-(MfeGly)_6_ GYGKKGRGDS-OH | 47 | 1642.68 |
| DfeGly6RGD | NH2-(DfeGly)_6_ GYGKKGRGDS-OH | 41 | 1750.62 |
| TfeGly4RGD | NH2-(TfeGly)_4_ GYGKKGRGDS-OH | 45 | 1580.41 |
| TfeGly6RGD | NH2-(TfeGly)_6_ GYGKKGRGDS-OH | 36 | 1858.57 |
| TfeGly8RGD | NH2-(TfeGly)_8_ GYGKKGRGDS-OH | Traces | 2136.72 |
| PfpGly4RGD | NH2-(PfpGly)_4_ GYGKKGRGDS-OH | 33 | 1779.59 |
| PfpGly6RGD | NH2-(PfpGly)_6_ GYGKKGRGDS-OH | 25 | 2158.61 |

The following pages report analytical information regarding individual pure peptides in the library. Chemical structures are reported. Purity after synthesis and purification was determined through analytical HPLC with DAD detection at 280 nm. HPLC system Chromaster 600 bar equipped with Kinetex C18 reverse-phase column. Solvent: (A) H2O + 0.1% TFA / (B) ACN + 0.1% TFA / gradient 10% (B) to 80% (B) over 18 min. Furthermore, corresponding mass spectra confirming the purity and identity of each peptide are reported.

## **Val4RGD**

**Figure S2:** Analytical information regarding Val4RGD

## **Val6RGD**

**Figure S3:** Analytical information regarding Val6RGD

## **Val8RGD**

**Figure S4:** Analytical information regarding Val8RGD

Mass spectra is not reported because ionization of this compound was not successful under the experimental conditions.

## **MfeGly6RGD**

**Figure S5:** Analytical information regarding MfeGly6RGD

## **DfeGly6RGD**

**Figure S6:** Analytical information regarding DfeGly6RGD

## **TfeGly4RGD**

**Figure S7:** Analytical information regarding TfeGly4RGD

## **TfeGly6RGD**

**Figure S8:** Analytical information regarding TfeGly6RGD

## **TfeGly8RGD**

**Figure S9:** Analytical information regarding TfeGly8RGD

## **PfpGly4RGD**

**Figure S10:** Analytical information regarding PfpGly4RGD

## **PfpGly6RGD**

**Figure S11:** Analytical information regarding PfpGly6RGD

# **RP-HPLC assay for estimation of hydrophobicity**

The protocol for the RP-HPLC assay was previously established by our group. Peptide samples were dissolved in 250 μL of a mixture of 10% (v/v) ACN in 80% (v/v) Milli-Q-water containing 0.1% TFA and filtered over a syringe filter with 0.2 μm pore size. The overall concentration of each sample was 0.2 mM. The retention times of all samples were determined on a C18 column (Capcell C18, 5 μm) using a Chromaster 600 bar DAD-System with UV detection at 280 nm. A linear gradient from 10 to 80% ACN + 0.1% TFA in 18 min was applied at room temperature and all experiments were performed in triplicates.

**Table ST3:** retention time of fluoropeptide conjugates library to assess their hydrophobicity

| Peptide | Retention time (min) |
| --- | --- |
| Val4RGD | 6.93 |
| **Val6RGD** | **8.21** |
| Val8RGD | 9.04 |
| **MfeGly6RGD** | **6.54** |
| **DfeGly6RGD** | **8.51** |
| TfeGly4RGD | 8.53 |
| **TfeGly6RGD** | **10.13** |
| TfeGly8RGD | 11.56 |
| PfpGly4RGD | 11.06 |
| **PfpGly6RGD** | **13.51** |

# **Peptide stock solution, concentration determination and sample preparation**

Peptide stock solutions were prepared by dissolving lyophilized peptide powder (10–15 mg) in 1,1,1,3,3,3-hexafluoropropan-2-ol [HFIP] (2 mL) and treatment for 15 min in an ultrasound- bath to dissolve preformed aggregates. An aliquot of 10 μL was evaporated and the dried peptide film dissolved in a 6 M guanidine hydrochloride (GndHCl) solution (pH 7.4), resulting into a dilution factor (DL) of 100. These samples were measured via UV detection at 280 nm by use of an Eppendorf BioPhotometer plus with semimicro-VIS Cuvettes (PMMA) 10 × 100 (Eppendorf, Hamburg, Germany). All UV spectra were baseline corrected with a reference spectrum of a sample containing solely buffer solution. The UV absorbance given through the fluorophore tyrosine (tyrosine) in the middle of the peptides at 280 nm was evaluated in triplicates. The concentration of each stock solution was calculated according to the Lambert-Beer law where the extinction coefficient was determined according to Pace et al.^30^ If not otherwise stated, all peptide samples were treated following this protocol before each measurement: an aliquot from the HFIP peptide stock solution was taken and evaporated. The dried peptide was then dissolved in phosphate buffer 10 mM pH 7.4 and vortexed (1 min), sonicated (5 min) and finally ultracentrifuged (1 min) at room temperature. The buffer was chosen to mimic physiological microenvironments.

# **Extensive CD analysis**

Circular dichroism experiments were performed using a Jasco J-810 spectropolarimeter fitted with a recirculating chiller (D-76227, Karlsruhe). Data were recorded using 0.1 mm Quartz Suprasil® cuvettes (Hellma) equipped with a stopper. Spectra were recorded at 37 °C from 190 to 250 nm at 0.2 nm intervals, 1 nm bandwidth, 4 s response time and a scan speed of 100 nm min^-1^. Baselines were recorded and were subtracted from the data. Each reported CD value represents the average of minimum three measurements. All (fluorinated)oligopeptides were studied at 1 wt% to induce a stable and soluble self-assembly.

**Figure S12:** CD spectra of amphiphilic oligopeptides Val6RGD, TfeGly6RGD and PfpGly6RGD at pH 7.4 (left) and of Val6RGD and TfeGly6 at pH 5.0 (right). The absence of self-assembled structures was confirmed by SAXS measurements (bottom right).

The result at pH 7.4 for Val6RGD (**Figure S12**) show a β-sheet conformation with the characteristic maximum at λ_max_ = 200 nm and negative band slightly shifted around λ_min_ = 218 nm. As demonstrated in previous work from our group, fluorine content increases the hydrophobic nature of amino acids while simultaneously decreasing the polarity promoting the beta-sheet formation.^31^ However, formation of canonical β-sheet was not observed for any of the fluoropeptides. Monofluorination induced random coil formation whereas the difluorinated sample exhibited limited solubility, preventing reliable acquisition and precluding definitive conclusions about its secondary structure in solution. (**Figure S13**).

**Figure S13:** CD spectra of amphiphilic oligopeptides MfeGly6RGD (left) and DfeGly6RGD (right) at 1 wt% concentration in phosphate buffer 10mM pH 7.4 recorded at 37 ^o^C.

For TfeGly6RGD, a non-canonical β-sheet-like secondary structure was detected with a weak maximum at λ_max_ = 190 nm and a deep negative band at λ_min_ =216 nm (π→π* transition) whose intensity and wavelength changed during incubation suggesting a time-dependent aggregation (**Figure S14**). Furthermore, no melting point was reached in the range 21 – 96 ^o^C confirming strong thermal stability since target oligopeptides retained their secondary structure during the experiment. Analyzing CD spectra of amphiphilic oligopeptides after annealing show an increase of intensity of the negative band at 219 nm for TfeGly6RGD, indicating a possibly higher degree of order reached upon cooling and decrease of the positive band at 200 nm for Val6RGD. PfpGly6RGD exhibited a similar absorption pattern to TfeGly6RGD, but the decrease of intensity is related to aggregation in solution observed as increase in solution turbidity, with a further negative band shift to λ_min_ = 214 nm (**Figure S12**). In correlation with the CD data, we suggest that a synergistic effect of intrinsic beta-sheet propensity, the fluorine-induced hydrophobicity by multiple incorporation of TfeGly and PfpGly and supramolecular structure formation are causing the folding pattern of the highly fluorinated peptides.

**Figure S14:** CD spectra of amphiphilic oligopeptides TfeGly6RGD (left) and Val6RGD (right) at 1 wt% at different time points and after annealing experiments.

# **Further Cryo-TEM images**

Perforated carbon film-covered microscopical 200 mesh grids (R1/4 batch of Quantifoil, MicroTools GmbH, Jena, Germany) were cleaned with chloroform and hydrophilized by 60 s glow discharging at 10 μA in a EMSCOPE SC500 before 4 μl aliquots of the peptide solution were applied to the grids. The samples were vitrified by automatic blotting and plunge freezing with a FEI Vitrobot Mark IV (Thermo Fisher Scientific Inc., Waltham, Massachusetts, USA) using liquid ethane as cryogen. The vitrified specimens were transferred to the autoloader of a FEI TALOS ARCTICA electron microscope (Thermo Fisher Scientific Inc., Waltham, Massachusetts, USA). This microscope is equipped with a high-brightness field-emission gun (XFEG) operated at an acceleration voltage of 200 kV. Micrographs were acquired on a FEI Falcon 3 direct electron detector (Thermo Fisher Scientific Inc., Waltham, Massachusetts, USA) using the 70 μm objective aperture at a nominal magnification of 28000, corresponding to a calibrated pixel size of 3.75 Å per pixel, respectively.

In order to get an insight into the morphology of the self-assembled particles formed by the new fluoro-peptide conjugates, cryo-TEM studies were performed on samples of amphiphilic oligopeptides in phosphate buffer 10 mM at pH 7.4. at 1 wt% concentration). Fluoropeptides TfeGly6RGD and PfpGly6RGD required a further dilution to 0.33 wt% due to their extremely enhanced contrast properties that interfered with the microscope image capture.

In-depth analysis, revealed that Val6RGD forms rigid, short, bilayer platelet morphologies characteristic of amyloid-like fibrils, with a thickness of approximately 18 nm (**Figure S15**).^32-35^

**Figure S15.** Representative cryo-TEM micrographies of Val6RGD indicating the formation of amyloid-like fibrils. The figure reports the further analysis of the fibrils width (“length”).

However, the higher fluorinated TfeGly6RGD revealed a more compact (thickness of approximately 8 nm) and elongated flexible fibrillar structure with the tendency of each fibrillar strand to aggregate into long and regular bundles. The flexibility of these fibrils is exhibited by helical twisting indicative of a hierarchical structural organization (**Figure S16**).

**Figure S16.** Representative cryo-TEM micrographies of TfeGly6RGD indicating the formation of longer, thinner and homogeneous filaments. The figure reports the further analysis of the fibrils and bundles widths (“length”).

Furthermore, the more sterically demanding sidechain of PfpGly lead to formation of aggregates with different morphologies, the fibrils in this case are shorter and exhibiting higher flexibility (**Figure S17**).

**Figure S17.** Representative cryo-TEM micrographies of PfpGly6RGD indicating the formation of shorter, more flexible fibrils. The figure reports the further analysis of the fibrils width (“length”).

Cryo-TEM of both mono- and difluorinated peptides together with the library of shorter conjugates Val4RGD, TfeGly4RGD and PfpGly4RGD show total absence of aggregates of any kind (**Figure S18**).

**Figure S18.** Representative cryo-TEM micrographies of the peptides excluded from further structural characterization: V4RGD, TfeGly4RGD an d PfpGly4RGD, V8RGD, MfeGly6RGD and DfeGly6RGD.

Furthermore, Val8RGD formed a hydrogel with a network density too high to allow reliable fiber analysis; therefore, these data are not reported (**Figure S18**).

# **SAXS data analysis**

SAXS measurements were performed in a flow-through capillary with a Kratky-type instrument (SAXSess, Anton Paar, Austria) at a temperature of 21 ± 1°C for all samples as delivered. The peptide concentration was 1 wt%. Before the measurement, the samples were vortexed for 30 seconds. The SAXSess has a low sample-to-detector distance of 0.309 m, making it suitable for investigating samples with low scattering intensity. Each sample was measured in its produced form for 600 seconds (60 measurements of 10 seconds each). Measurements were adjusted by subtracting the scattering of the buffer. The scattering vector *q* is defined in terms of the scattering angle 2θ and the wavelength *λ* of the radiation (*λ* = 0.154 nm): thus *q* = 4 π/*λ* sin(*θ*). Deconvolution (slit length desmearing) of the SAXS curves was conducted using SAXS-Quant software (Anton Paar, Austria). Data evaluation utilized SASfit software.^36^

SAXS measurements were initially conducted on samples of Val6RGD and TfeGly6RGD at pH values of 7.4 and 5. The scattering patterns of both samples at the same pH value are clearly similar; however, they differ significantly at different pH levels, as shown in the left and right panels of (**Figure S19**). The models that best matched the data at pH 7.4 were long filaments featuring an elliptical cross-section, while random coil structures were observed at pH 5. Best-fit values for the short axis of the cylindrical cross-section are *a* = 3.94 ± 0.15 nm for Val6RGD and *a* = 2.40 ± 0.07 nm for TfeGly6RGD. The cylinder lengths were held constant at 100 nm to avoid ambiguous results. The random coil structures are characterized by a radius of gyration of *R*_g_ = 1.10 ± 0.19 nm for Val6RGD and *R*_g_ = 1.33 ± 0.09 nm for TfeGly6RGD. An inspection of the curves reveals that the intensity of TfeGyl6RGD displays higher intensities than that of Val6RGD. This finding can be attributed to the larger scattering contrast of TfeGyl6RGD compared to Val6RGD due to the higher electron density of fluorine relative to hydrogen.

**Figure S19.** (Left) SAXS patterns of Val6RGD and TfeGly6RGD at pH 7 (gray and green solid lines) alongside the corresponding model curves, which are based on the structural model of a long cylinder with an elliptical cross-section (solid red and dashed blue lines). (Right) SAXS pattern of Val6RGD and TfeGly6RGD at pH 5 (gray and green solid lines) alongside the corresponding model curves, which are based on the structural model of a random coil (solid red and dashed blue lines).

Next, we compare the SAXS patterns of the samples with increasing fluorine content along Val6RGD, including MefGly6RGD, DfeGly6RGD, TfeGly6RGD, and PfpGly6RGD at pH 7.4 (see **Figure 5C**). In addition to the data for Val6RGD and TfeGly6RGD, the curve of PfpGly6RGD is best interpreted as a cylinder with an elliptical cross-section with a short semi-axis of *a* = 2.58 ± 0.06 nm and a long semi-axis of *b* = 6.22 ± 0.36 nm. However, unlike Val6RGD and TfeGly6RGD, for which the cylinder length exceeds the upper measurement limit of *d*_max_ = 40 nm, the cylinder length for PfpGly6RGD is L = 20 ± 2 nm. The curves of the remaining samples are accurately described by random coil structures with *R*_g_ = 0.94 ± 0.06 nm for MefGly6RGD and *R*_g_ = 1.10 ± 0.07 for DfeGly6RGD.

Finally, measurements were taken three days after sample preparation to investigate whether the cylindrical structures changed over time. The result is summarized in **Figures S20** and **S21** show that the cross-sectional area of TfeGly6RGD slightly shrinks within the measurement uncertainty, whereas for Val6RGD and PfpGly6RGD, the cross-section areas slightly increase from day 1 to day 3. Additionally, the cylinder length of PfpGly6RGD increases from 20 (day 1) to 40 nm (day 3).

We furthermore investigated the question whether the random coil structures formed by MfeGly6RGD and DfeGly6RGD at pH 7.4 and Val6RGD and TfeGly6RGD at pH 5 stem from single peptide molecules. Here, we reference the work of Kohn et al., who investigated the random-coil behavior and dimensions of chemically unfolded proteins.^37^ Therein, the radius of gyration is given as:

$$R_{g}\text{=}R_{0}N^{\nu},$$

where *R*_0_ is a persistence length of 0.1929 ±0.0271 nm, N is the number of monomers, and $\nu$ is an exponent, which is 1/2 for a random coil. In a “good” solvent where the coil expands, $\nu$ is larger than 0.5 (e.g., 3/5) and in a “poor” solvent, where the coil collapses $\nu$ is smaller than 0.5 (e.g., 1/3). We tested for deviation of the coil conformation from a random coil structure by employing the generalized Gaussian coil model.^38,39^ However, within the uncertainty of the data, there is no evidence for deviation of the random-coil structure, i.e., was indicated (not shown). Therefore, with $\nu$ =1/2 and *N* = 15, we could expect a theoretical value of *R*g = 0.75 ± 0.10 nm. The measured values and 1.10 ± 0.19 nm (Val6RGD), 0.94 ± 0.06 nm (MfeGly6RGD), 1.10 ± 0.07 nm (DfeGly6RGD) and 1.33 ± 0.09 nm (TfeGly6RGD) are slightly larger than the expected value. Likely, the peptides in our study require slightly more space than typical proteins per amino acid unit. Nevertheless, the radii of gyration values indicate the presence of non-interaction peptides. Larger aggregates in these samples can be excluded because the scattering intensity scales with the square of the aggregate's volume. Therefore, even small amounts of aggregates will be obtained as a steep increase in the scattering curves at low q-values.

**Figure S20.** Short and long semi-axis (*a* and *b*) of the cylindrical cross-section of samples Val6RGD. Data are reported as Mean ± SD, n = 3

**Figure S21.** The cross-section area of samples Val6RGD, TfeGly6RGD, and PfpGly6RGD was determined on day 1 and day 3 after sample preparation. Data are reported as Mean ± SD, n = 3

# **Thioflavin T (ThT) fluorescence assay for amyloid detection**

The ability of amphiphilic fluoropeptides to form cross-β-sheet structures, especially at high degree of fluorination has been recently reported by our group.^20^ However, for notable exception of the non-fluorinated Val6RGD, none of the fluoropeptides folds accordingly. This finding was confirmed by Thioflavin T (ThT) fluorescence spectroscopy. The ThT dye displays a strong fluorescence upon binding to amyloid morphologies caused by rotational immobilization of the aromatic rings, leading to an increase in fluorescence emission with a maximum at 485 nm. A suitable protocol for this assay was recently published by our group and re-adapted to suit operative conditions.^40^ Aliquots of peptide HFIP-stock solutions were dried and then redissolved in 10 mM phosphate buffer with addition of 20 μM Thioflavin T (overall pH 7.4). The buffer containing ThT was previously filtered over a nylon syringe filter with 0.2 μm pore size. After dissolution, the sample was sonicated for 30 s, transferred on a BRAND® microplate (size: 96 wells, color: black; Sigma-Aldrich), sealed to prevent evaporation and placed in an Infinite® M Nano+ plate reader (Tecan Deutschland GmbH, Crailsheim, Germany). ThT fluorescence (ex = 420 nm, em = 485 nm, Z-position: 15 173 nm [manual], gain: 80 [manual], lag time: 0 μs, integration time: 20 μs) was measured after 24 h incubation at 37 °C. The fluorescence intensity at 485 nm was normalized with respect to the negative control solely containing buffer (set as FL_int_ 1.0).

Samples of the fluoropeptide conjugates in a wide range of concentration (0.02 – 1 wt%) were monitored for 24 hours to evaluate the concentration-dependent kinetics of the amyloid formation process. Significative fluorescence intensity (FL) was observed in the range 0.1 – 0.25 wt% for none of the library members. Furthermore, fluoropeptides did not show any substantial fluorescence enhancement either in the 0.33 – 1 wt% range indicating no formation of amyloid-like aggregates. In contrast, Val6RGD shows strong concentration-dependent fluorescence intensity up to a dramatic 180-fold enhancement at 1 wt% compared to the control sample (sole ThT dye in buffer, Fluorescence intensity FL_485nm_ = 1.0) indicating amyloid formation. Kinetic curves (**Figure S22**), indicate time-dependent formation of amyloid morphologies. Amyloid formation is observed at concentrations ≥ 0.1 wt%, with a lag time of approximately 6h from 0.25 wt% and almost immediate formation at 1 wt%.

**Figure S22:** Thioflavin T experiments of Val6RGD (0.02 – 1 wt%) in phosphate buffer 10mM + 20 μM ThT dye at 37^o^C for 24 h.

# **Extensive MD analysis**

**Simulation model preparation:**

The initial structure of the polypeptide Val6RGD was built using AmberTools.^41^ For the fluorinated polypeptide TfeGly6RGD, the CHARMM36m^42^ parameters along with our recently optimized CHARMM-based^42,43^ force field parameters for fluoro-ethylene glycines^20^ were used.

**Simulation system setup:**

Each simulation system contained 30 identical peptide chains initially placed randomly in a cubic simulation box using PACKMOL. Peptides were solvated with explicit TIP3P water molecules and neutralized with NaCl at a final ionic strength of 0.15 M. Four distinct protonation/charge states were considered:

1. Val6RGD LysH+, N-terminal NH3+
2. Val6RGD Lys0, N-terminal neutral
3. Val6RGD LysH+, N-terminal neutral
4. TfeGly6RGD LysH+, N-terminal neutral

**Molecular dynamics simulations:**

All simulations were performed using GROMACS 2025.^44^After energy minimization, each system was equilibration in NVT and NPT ensembles, followed by production simulation in the NPT ensemble. Production simulations were run for 2 μs in each condition. Protein interactions were described using CHARMM36m, with TIP3P water.^45^ Long-range electrostatics were treated with the particle mesh Ewald method (cutoff 12 Å)^46^, and van der Waals interactions were smoothly switched to zero between 10–12 Å. Covalent bonds involving hydrogen were constrained with LINCS^47^, allowing a 2 fs integration step. Temperature was maintained at 310 K with the velocity-rescaling thermostat^48^, and pressure at 1 bar with the Parrinello–Rahman barostat.^49^

**Analysis:**
Trajectory analyses were performed with MDAnalysis,^50,51^ MDTraj^52^, and in-house Python scripts. Structural and dynamical properties included: secondary structure content (DSSP)^53^, radius of gyration, peptide–peptide contact maps, and cluster size distributions.

**Dataset analysis:**

Under mildly acidic conditions, both V6RGD and TfeGly6RGD displayed a loss of ordered secondary structure. Since lysine side chains are expected to remain largely protonated at both pH 7.4 and pH 5, this pH response is not attributed to lysine protonation alone. Rather, acidification changes the overall ionization, counterion association, and hydration balance of the amphiphilic assemblies, including partial protonation of carboxylate groups in the C-terminal/RGD region. This altered electrostatic environment destabilizes the ordered supramolecular state, consistent with the CD and SAXS data in **Figure 4**, which show loss of ordered secondary structure and absence of assembled structures under acidic conditions.

To assess how protonation states and counterions modulate the mechanism of peptide self-assembly, we conducted molecular dynamic simulations of 30-chain system containing of Val6RGD and its fluorinated analogue TfeGly6RGD under four distinct conditions: (I) Val6RGD LysH⁺/N-ter NH₃⁺, (II) Val6RGD Lys⁰/N-ter neutral, (III) Val6RGD LysH⁺/N-ter neutral and (IV) TfeGly6RGD LysH⁺/N-ter neutral. The protonation states considered in the simulations were chosen to evaluate the influence of peptide charge distribution on aggregate formation and compaction. Accordingly, these models should not be interpreted as direct molecular representations of the experimental pH 7.4 and pH 5 conditions, but rather as a means to assess the charge sensitivity of the assembly process. Notably, only the fully protonated Val6RGD LysH⁺/N-ter NH₃⁺ condition produced multiple aggregates in the simulations after 1 μs, whereas all other states yielded a single dominant cluster (**Figure S23**). These preliminary results suggest that N-terminal protonation disrupts compact packing and promotes the formation of multiple coexisting assemblies. In contrast, neutralization or the presence of trifluorinated analogue (TFA) promotes consolidation into a single aggregate. Having established these distinct aggregation patterns, we then examined the **structural content of the resulting assemblies.**

**Figure 23: Aggregate formation under different protonation states.** Violin plot showing the plateau number of aggregates formed after 1 μs of simulation for 30-chain systems under four conditions: (i) Val6RGD LysH⁺, N-terminal NH₃⁺, (ii) Val6RGD Lys⁰, N-terminal neutral, (iii) Val6RGD LysH⁺, N-terminal neutral, and (iv) TfeGly6RGD LysH⁺, N-terminal neutral. Multiple aggregates persist only under the fully protonated LysH⁺/NH₃⁺ state, whereas all other conditions yield a single dominant aggregate.

Across all conditions, peptides progressively acquired β-sheet content over the 2 μs simulation time (**Figure S24**). The average β-sheet fraction stabilized near 28% for LysH⁺/N-ter NH₃⁺ and Lys⁰/N-ter neutral, with slightly reduced values for the TfeGly systems (22%). Heat maps of individual chains revealed highly heterogeneous folding dynamics: whereas Val6RGD rapidly adopted stable β-structure, TfeGly6RGD remained largely ­disordered throughout (**Figure 7A**). This chain-to-chain variability mirrors experimental circular dichroism (**Figure 4**) observations of incomplete β-sheet conversion in fluorinated peptide systems and confirms Val6RGD amyloid fibril formation (**Figure 5B**). While β-sheet content provides insight into secondary structure formation, it does not directly reflect the overall shape and compactness of the assemblies. Cryo-TEM and SAXS measurements (**Figure 5 A, C and D**) highlight substantial differences in the packing arrangements of Val6RGD compared to its fluorinated analogs indicating tighter packing organization at higher degrees of fluorination. We therefore quantified the global dimensions of the aggregates through radius of gyration (Rg) analysis (**Figure S25**) where assemblies formed under LysH⁺/N-ter neutral conditions remained expanded for hundreds of nanoseconds before reaching compaction. Coherently with the other experimental findings, the final plateau Rg distributions revealed significantly tighter packing for TFeGly6RGD. This behavior is consistent with the flexible fibrillar morphologies observed by cryo-TEM and the compact cross-sections identified by SAXS.

**Figure S24: Time evolution of β-sheet content.** Top: Average β-sheet content across all chains over the course of 2 μs for the four protonation conditions. Shaded areas indicate standard deviations. Bottom: Heat maps of chain-resolved β-sheet content for each system.

To elucidate the molecular mechanism underlying the fluorine-induced β-sheet disruption and structural compaction inter-chain contacts (**Figure S26**) were studied. Detailed contact analysis showed that TfeGly substitution altered the balance between backbone and side-chain interactions. Whereas Val6RGD systems exhibited frequent backbone–side chain and side chain–side chain contacts, TfeGly6RGD assemblies displayed markedly fewer side-chain interactions. Instead, stabilization relied more heavily on backbone–backbone contacts. This is corrobated by chord diagrams (**Figure S27**), which highlight reduced cross-residue diversity of interactions in fluorinated assemblies. Notably, the increased packing density did not lead to reduced solvent accessibility. Instead, TfeGly6RGD assemblies exhibited higher hydration levels than the control Val6RGD (**Figure 7B**). Specifically, hydration analysis revealed an increased number of water molecules within 5 Å of the peptide backbones in TfeGly6RGD (**Figure S28**). This counterintuitive combination of dense packing and enhanced solvation aligns with experimental observations that fluorination reduces amyloid-like aggregation propensity and promotes soluble, dynamic, yet tightly packed fibrillar architectures. Overall, simulation results provide a mechanistic explanation of how protonation and fluorination shape peptide assembly. In fact, protonation of the N-terminus disrupts compact packing and favors fragmented aggregates. Furthermore, the balance of backbone–backbone, side chain–backbone, and side chain-side chain interactions plays a critical role in defining the assembly pathway. In the fluorinated variants, these interactions do not support the cooperative stabilization of β-sheets and, together with enhanced hydration, promoted more disordered packing. By contrast, the wild-type sequence achieves efficient dehydration of the hydrophobic core and maintains backbone–backbone, backbone-side chain contacts that favored the formation of more ordered, β-sheet–rich assemblies.

**Figure S25: Structural organization and compaction of peptide assemblies.** Representative snapshots of assemblies at the end of 2 μs showing β-sheet-rich (yellow) vs. disordered (pink) structures. Middle: Time evolution of the radius of gyration (Rg) comparing Val6RGD LysH⁺/neutral and TfeGly6RGD LysH⁺/neutral conditions. Bottom: Plateau Rg distributions (t ≥ 1 μs).

**Figure S26:** **Inter-chain contact propensities.** Violin plots of backbone–backbone (bb–bb), backbone–side chain (bb–sc), and side chain–side chain (sc–sc) contact fractions for Val6RGD vs. TfeGly6RGD assemblies.

**Figure S27:** **Residue-level interaction networks.** Chord diagrams summarizing inter-residue contacts for Val (left) and TfeGly (right) systems. While both maintain contacts with Lys, Tyr, and Gly residues.

**Figure S28:** **Solvation of β-sheet cores.** Representative structures of Val6RGD LysH⁺/neutral (left) and TfeGly6RGD LysH⁺/neutral (right) assemblies, with water molecules within 5 Å of peptide backbones shown as gray density. Center: Violin plots of water coordination per site.

# **Peptide digestion assay (Real time monitoring)**

For real-time monitoring of enzymatic digestion, peptides Val6RGD and TfeGly6RGD were dissolved in 10 mM phosphate buffer, pH 7.4 (100 μL) at a 5-fold concentration of 0.02 wt% to prevent self-assembly and gently mixed to obtain a homogeneous mixture. After that, 100 μL of elastase (4 μM), dissolved in 10 mM phosphate buffer pH 7.4 was added and the samples were gently mixed for 5 s. The final reaction mixture comprised 0.01 wt% peptide and 2 μM enzyme, respectively. All samples were incubated at 30 ^o^C over a period of 3 h. Aliquots of 40 μL were taken at fixed time points and quenched with 40 μL of a solution of 5% TFA in water containing 50 μM NH_2_-Gly-Tyr-OH as reference. Peptide degradation was monitored by HPLC analysis, and the remaining amounts of substrates were calculated by comparison to the reference signal. Proteolysis experiments were run in triplicates.

First, a calibration of the control peptide Val6RGD with three different enzyme concentrations (4 μM, 0.4 μM, 0.04 μM) was performed by a reversed-phase HPLC assay over three hours (**Figure S29 A**). The control peptide remained almost untouched at 0.004 μM enzyme concentration. Similarly, at an enzyme concentration of 0.4 μM, approximately 70% of the peptide remained in solution after 3 hours. On the contrary, the peptide showed a good degradation profile at a 4 μM enzymatic concentration (**Figure S29 B**) Therefore, an enzyme concentration of 4 μM was chosen for the real-time monitoring of enzymatic digestion. A reversed-phase HPLC assay of the respective peptides at a concentration of 0.02 wt% was performed with a Gly-Tyr reference in the presence of elastase (4 μM) over three hours. It should be mentioned that the HPLC signals of the target peptides showed significantly low intensities (besides for Val6RGD) compared to the reference (**Figure S29, D**). This could indicate that the respective peptide tends to aggregate, even at this low concentration. Regardless, the fluorinated peptide TfeGly6RGD was recognized and degraded by the enzyme (**Figure S29 C**).

Proteolysis experiments were performed to evaluate the susceptibility of the compounds to enzymatic degradation. The cleavage products were not monitored, as the aim of the experiment was to assess whether degradation occurred rather than to identify specific cleavage sites. This approach was justified given that the reaction mechanism and cleavage preferences of elastase are well established. In addition, the low compound concentrations employed, chosen to prevent aggregation, were below the detection limit for mass spectrometric analysis of cleavage products.

**Figure S29**: A. Structures of the target peptides, Gly-Tyr reference, and porcine elastase (1LVY), B. Enzymatic degradation profile of the calibration using Val6RGD in the presence of elastase at three different concentrations, C. Enzymatic degradation profile of Val6RGD and TfeGly6RGD. D. HPLC chromatograms of the protease assay for degradation of TfeGly6RGD. Data are reported as Mean ± SE, n = 3.

# **Carrier properties: pyrene encapsulation experiments**

To elucidate the self-assembly behaviour of the new fluoropeptide conjugates as a function of fluorine content and hydrophobic chain length peptides were directly dissolved in buffer and the aggregation behaviour was monitored by fluorescence spectroscopy using pyrene as a hydrophobic probe.^54,55^ The fluorescence intensity of pyrene increases with the elevated concentration of amphiphilic peptide indicating the formation of a core-shell structure and the transfer of pyrene from the hydrophilic outer medium (in which is not soluble) into the hydrophobic inner core of the architecture formed.

Aliquots of a pyrene solution (6 µM in acetone, 150 µL) were added to plastic tubes, and the solvent was allowed to evaporate with gentle nitrogen stream. Subsequently amphiphilic oligopeptides were dissolved in phosphate buffer 10 mM pH 7.4 and added to the tube containing pyrene. The samples were incubated for 1 h at 37 °C to reach pyrene solubilization equilibrium. Fluorescence excitation was performed at 334 nm, and emission spectra were recorded from 360 to 600 nm. Experiments were performed in triplicate.

**Figure S30.** FL-based pyrene encapsulation assay for TfeGly6RGD (left) and Val6RGD (right). An enhancement in fluorescence intensity relates to solubilization of pyrene in a highly hydrophobic environment. Each curve represents the average of three independent measurements.

No significant fluorescent signal was measured in the concentration range of 0.02 - 0.05 wt% indicating no core-shell structure formation resulting in no solubilization of pyrene in a hydrophobic environment (**Figure S30**). However, in the range of 0.1 – 1 wt% a dramatic enhancement in fluorescence intensity was observed for Val6RGD and TfeGly6RGD that even exhibited the highest overall encapsulation efficiency at 1 wt%. In contrast, the packing pattern of MfeGly6RGD, DfeGly6RGD and PfpGly6RGD are inhibiting the capacity of the oligopeptide to accommodate the pyrene molecules since no significant fluorescent signal was detected across the whole concentration range.

**Figure S31.** Fluorescence spectra of TfeGly6RGD and Val6RGD with (positive control) and without pyrene (negative control) and spectra of sole pyrene in buffer as a further negative control.

To confirm that the observed fluorescence arises from pyrene–peptide interactions rather than intrinsic peptide fluorescence, emission spectra were recorded for samples of TfeGly6RGD and Val6RGD in the presence and absence of pyrene (**Figure S31**). Furthermore, a spectrum of pyrene in buffer was recorded as additional negative control. The results indicate that the fluorescence signal is specific to the interaction with pyrene.

# **Doxorubicin drug loading in vitro release monitoring method**

Drug loading was performed as follows: 10 mg of peptides and 2 mg of doxorubicin were dissolved in 5 mL of DMSO. The solution was transferred to a dialysis tube (MWCO 1 KDa) and subjected to dialysis against distilled H_2_O for 24 h; during this period of time the outer medium was exchanged every 4 hours to remove DMSO and unloaded drug. Subsequently, the in vitro release behavior was monitored at physiological temperature (37 ^o^C) by directly immerging the tube into 10 mL of fresh distilled water at pH 7.4 or 5.0. At fixed time points aliquots of the outer medium were sampled and fresh water was exchanged. Each aliquot was transferred on a BRAND® microplate (size: 96 wells, color: black; Sigma-Aldrich), and placed in an Infinite® M Nano+ plate reader (Tecan Deutschland GmbH, Crailsheim, Germany). The amount of doxorubicin released from the respective peptides was measured at the emission wavelength of 556 nm and excitation wavelength of 490 nm. The average value of three independent experiments was collected and the cumulative DOX release was calculated as follows:

$$\text{ }Cumulative\text{ }release\text{ }\left( \text{\%} \right)\text{=}\left( \frac{M_{t}}{M_{\infty}} \right)\text{*}100$$

Where M_t_ is the amount of DOX released from the peptide at time t and M_∞_ is the amount of DOX loaded in the peptide. To determine M_∞,_ the loaded peptides after performing drug release were lyophilized and the dried sample was redissolved in 1 mL of milli q water. The residual doxorubicin was determined through fluorescence spectroscopy as above. M_∞_ is calculated as the sum of the drug released by the sample and the residual drug.

# **Cell experiments**

**All cell experiments were conducted according to German genetic engineering laws and German biosafety guidelines in the laboratory (safety level 2)**.

## **Cytotoxicity test - live/dead assay**

Cell viability was determined using a Live-Dead Viability Kit, consist of 4 mM Calcein and 2 mM Ethidium Homodimer-1 (Thermo Fisher Scientific; Art. L3224) according to the manufactor´s instructions. A549, HeLa and McF7 cells were obtained from Leibniz-Institut DSMZ - Deutsche Sammlung von Mikroorganismen und Zellkulturen GmbH and cultured in DMEM Medium supplemented with 10% (v/v) FBS, 100 U/mL penicillin and 100 μg/mL streptomycin. Cells were seeded in 8-well slides (Ibidi GmbH, Gräfelfing, Germany) at a density of 5 x 10^4^ cells/ml and incubated overnight for cell attachment. Afterwards negative control (DMEM Medium) and samples (directly solved in DMEM Medium) were added to cells at 3 different concentrations and incubated for another 24 h at 37 °C and 5% CO_2._ Before adding the staining solution, cells for dead controls were treated with 70% EtOH for approximately 1 h at 37 °C and 5% CO_2._ For Preparation of staining solution 20 µl of 2 mM Ethidium Homodimer-1 (final: 4 µM) and 5 µl of 4mM Calcein (final: 2 µM) were thoroughly mixed in 10 mL PBS buffer. Samples, Medium and EtOH were discarded, cells were covered with approximately 300 µl fresh Live-Dead-solution and incubated for 30 – 45 minutes at room temperature in the dark. To avoid any background fluorescence cells were washed once with PBS buffer and covered again with DMEM Medium. Fluorescence (Emission at 517 nm for Calcein, 617 nm for Ethidium Homodimer-1, Excitation Wavelength Range 494 - 528 nm) was detected and images were taken by an inverted Leica TCS SP8 (Leica, Wetzlar, Germany) with 5 x objective lens and Leica LAS X Software for image processing in sequential mode.

**Figure S32:** (Top) Cell viability (%) of HeLa, A549 and McF7 cells treated with amphiphilic oligopeptides Val6RGD and TfeGly6RGD showing the effect of increasing fluorination degree at different concentrations. (Bottom) representative CLSM images of the cell cultures after the experiment. Data are reported as Mean ± SE, n = 6.

The cell lines were selected as standard models for RGD-tagged peptides because of their well-established integrin receptor expression. We conducted a confocal scanning laser microscopy (CLSM)-based live/dead assay leaving Val6RGD and TfeGly6RGD at a certain concentration (0.001 wt% – 0.1 wt%) directly in contact with the cell medium (**Figure S32**). The cell viability was determined after 24 hours of incubation time analyzing the fluorescence micrographies with a fast and open-source macro for Fiji recently published by Kerkhoff et al.^56^

No cytotoxic effect was detected up to 0.01 wt% (concentration below the self-assembly threshold) regardless of the fluorine content. However, reaching the self-assembly threshold 0.1 wt% concentration we detected a considerable cytotoxic effect for Val6RGD across all cell lines tested. Considering the heterogeneous growth of McF7 cells, further experiments were limited to A549 and HeLa cells.

**Figure S33.** Cell viability (%) of A549 (top) and HeLa (bottom) cells treated with amphiphilic oligopeptides MfeGly6RGD, DfeGly6RGD and PfpGly6RGD showing the effect of intermediate and higher fluorination degree at different concentrations. Data are reported as Mean ± SE, n = 3.

The effect of progressively increasing fluorine content was examined by evaluating the cytotoxicity of MfeGly6RGD and DfeGly6RGD, which exhibit intermediate degrees of fluorination, and PfpGly6RGD, which exhibits a higher degree of fluorination (**Figure S33**). The implementation of MfeGly drastically reduced toxicity bringing cell viability up to almost 100%. A further increase in fluorine content led to a slight decrease in cell viability. However, values upholded the established toxicity threshold (cell viability below 80%) on A549 and HeLa thereby confirming the biocompatibility of the new fluoropeptide conjugates for potential cellular applications. At the highest concentration tested, a slightly toxic effect was detected for PfpGly6RGD and only in case of HeLa. Results on cytotoxic properties of amphiphilic oligopeptides 0.1 wt% on A549 and HeLa were then summarized (**Figure S34**).

**Figure S34.** Summarized cell viability (%) of A549 (left) and HeLa (right) cells treated with amphiphilic oligopeptides Val6RGD, MfeGly6RGD, DfeGly6RGD, TfeGly6RGD and PfpGly6RGD showing the effect of intermediate and higher fluorination at operative concentration. Data are reported as Mean ± SE, n = 3.

## **Intracellular uptake**

The cellular uptake of DOX-labeled peptides was analyzed using an inverted Leica TCS SP8 (Leica, Wetzlar, Germany) with 63 x oil-immersion objective lens and Leica LAS X Software for image processing in sequential mode. A549 cells were obtained from Leibniz-Institut DSMZ and cultured in DMEM Medium supplemented with 10% (v/v) FBS, 100 U/mL penicillin and 100 μg/mL streptomycin at 37 °C and 5% CO_2_. Cells were seeded in 8-well slides (Ibidi GmbH, Gräfelfing, Germany) at a density of 5 x 10^4^ cells/ml and incubated overnight for cell attachment. Afterwards sample (solved in deionized water) was added to cells at 3 different concentrations. After incubation of 20h and 2h respectively cells were stained with Hoechst 33342 dye (1 µg/mL, Life Technologies GmbH, Darmstadt, Germany), washed 3 times with PBS and covered with fresh DMEM cell culture medium. Hoechst 33342 was excited with 405 nm UV Laser and Doxorubicin was excited using the 561 nm Laser. The Emission detection of Hoechst 33342 was performed between 410-485 nm (hybrid detector) and Doxorubicin between 496nm - 629nm (hybrid detector). Following results refer to peptide concentration 0.1 wt%.

**Figure S35:** CSLM-based cellular uptake assay for TfeGly6RGD (top) and Val6RGD (bottom).

Fluorescence imaging clearly demonstrates successful intracellular delivery of doxorubicin (labeled in bright pink) into A549 cells. This targeted delivery is mediated by the interaction between RGD-tagged peptides and integrins expressed on the cell membrane, which facilitates the recognition and uptake. DAPI-stained images of cells treated with DOX-loaded peptides reveal the distribution of DOX around membrane with progressive penetration through the nucleus observed after 24 hours (**Figure 35 h, p**). Notably, a reduction of DAPI fluorescence intensity (light blue) is evident in treated samples (**Figure 35 e, m**) compared to unloaded controls (**Figure 35 a, i**), consistent with nuclear accumulation of DOX accumulation and its known interactions with chromatin, including DNA intercalation and potential chromatin condensation.^57^ Combining the pH responsive self-assembly, extremely low toxicity whereas enhanced loading capabilities and receptor-specific targeting, TfeGly6RGD shows great potential as drug-carrier for tumor-targeting therapeutics.

**Figure S36.** CLSM images of A459 cells incubated with DOX-loaded TfeGly6RGD for 2h (top) and 20h (bottom).

**Figure S37.** CLSM images of A459 cells incubated DOX-loaded Val6RGD for 2h (top) and 20h (bottom).

**Figure S38.** CLSM images of A459 cells incubated with TfeGly6RGD (no DOX) for 2h (top) and 20 h (bottom).

**Figure S39.** CLSM images of A459 cells incubated with Val6RGD (no DOX) for 2h (top) and 20 h (bottom).

**Figure S40.** CLSM images of A459 cells recorder after 20 h in absence of peptide (negative control).

**Β-sheet orientation**

**Figure S41.** Time evolution of β strand contact orientation in the MD simulations. β structured residues were identified using DSSP, after which paired β strand contacts were detected from backbone contacts and classified as parallel, antiparallel, or ambiguous from the relative N to C orientation of Cα backbone vectors. The plot shows that parallel and antiparallel contacts coexist throughout the trajectories

**References**

[27]	T. T. Romoff, B. G. Ignacio, N. Mansour, A. B. Palmer, C. J. Creighton, H. Abe, H. Moriwaki, J. Han, H. Konno, V. A. Soloshonok, “Large-Scale Synthesis of the Glycine Schiff Base Ni(II) Complex Derived from (S)- and (R)-N-(2-Benzoyl-4-chlorophenyl)-1-[(3,4-dichlorophenyl)methyl]-2-pyrrolidinecarboxamide” Organic Process Research & Development 24, (2020): 294–300.

[28]	J. Han, R. Takeda, X. Liu, H. Konno, H. Abe, T. Hiramatsu, H. Moriwaki, V. A. Soloshonok, “Preparative Method for Asymmetric Synthesis of (S)-2-Amino-4,4,4-trifluorobutanoic Acid” Molecules 24, (2019).

[29]	J. Leppkes, N. Dimos, B. Loll, T. Hohmann, M. Dyrks, A. Wieseke, B. G. Keller, B. Koksch, “Fluorine-induced Polarity Increases Inhibitory Activity of BPTI towards Chymotrypsin” RSC Chemical Biology 3, (2022): 773–782.

[30]	C. N. Pace, F. Vajdos, L. Fee, G. Grimsley, T. Gray, “How to Measure and Predict the Molar Absorption Coefficient of a Protein” Protein Science 4, (1995): 2411–2423.

[31]	U. I. M. Gerling, M. Salwiczek, C. D. Cadicamo, H. Erdbrink, C. Czekelius, S. L. Grage, P. Wadhwani, A. S. Ulrich, M. Behrends, G. Haufe, et al., “Fluorinated Amino Acids in Amyloid Formation: A Symphony of Size, Hydrophobicity and α-Helix Propensity” Chemical Science 5, (2014): 819–830.

[32]	I. W. Hamley, “Peptide Fibrillization” Angewandte Chemie International Edition 46, (2007): 8128–8147.

[33]	O. S. Makin, L. C. Serpell, “Structures for Amyloid Fibrils” FEBS Journal 272, (2005): 5950–5961.

[34]	M. Sunde, C. Blake, “The Structure of Amyloid Fibrils by Electron Microscopy and X-ray Diffraction” Advances in Protein Chemistry 50, (1997): 123–159.

[35]	A. Bertolani, L. Pirrie, L. Stefan, N. Houbenov, J. S. Haataja, L. Catalano, G. Terraneo, G. Giancane, L. Valli, R. Milani, et al., “Supramolecular Amplification of Amyloid Self-Assembly by Iodination” Nature Communications 6, (2015): 7574.

[36]	I. Bressler, J. Kohlbrecher, A. F. Thunemann, “SASfit: A Tool for Small-Angle Scattering Data Analysis Using a Library of Analytical Expressions” Journal of Applied Crystallography 48, (2015): 1587–1598.

[37]	J. E. Kohn, I. S. Millett, J. Jacob, B. Zagrovic, T. M. Dillon, N. Cingel, R. S. Dothager, S. Seifert, P. Thiyagarajan, T. R. Sosnick, et al., “Random-Coil Behavior and the Dimensions of Chemically Unfolded Proteins” Proceedings of the National Academy of Sciences of the United States of America 101, (2004): 12491–12496.

[38]	B. Hammouda, “Small-Angle Scattering From Branched Polymers” Macromolecular Theory and Simulations 21, (2012): 372–381.

[39]	B. Hammouda, “Form Factors for Branched Polymers with Excluded Volume” Journal of Research of the National Institute of Standards and Technology 121, (2016): 139–164.

[40]	S. Chowdhary, J. Moschner, D. J. Mikolajczak, M. Becker, A. F. Thünemann, C. Kästner, D. Klemczak, A.-K. Stegemann, C. Böttcher, P. Metrangolo, et al., “The Impact of Halogenated Phenylalanine Derivatives on NFGAIL Amyloid Formation” ChemBioChem 21, (2020): 3544–3554.

[41]	S. Khuvis, K. Tomko, S. R. Brozell, C.-C. Chen, H. Subramoni, D. K. Panda, “Optimizing Amber for Device-to-Device GPU Communication” Practice and Experience in Advanced Research Computing, (2023).

[42]	J. Huang, S. Rauscher, G. Nawrocki, T. Ran, M. Feig, B. L. de Groot, H. Grubmüller, A. D. MacKerell Jr., “CHARMM36m: An Improved Force Field for Folded and Intrinsically Disordered Proteins” Nature Methods 14, (2017): 71–73.

[43]	K. Vanommeslaeghe, E. Hatcher, C. Acharya, S. Kundu, S. Zhong, J. Shim, E. Darian, O. Guvench, P. Lopes, I. Vorobyov, et al., “CHARMM General Force Field: A Force Field for Drug-Like Molecules Compatible with the CHARMM All-Atom Additive Biological Force Fields” Journal of Computational Chemistry 31, (2010): 671–690.

[44]	M. J. Abraham, T. Murtola, R. Schulz, S. Páll, J. C. Smith, B. Hess, E. Lindahl, “GROMACS: High Performance Molecular Simulations through Multi-Level Parallelism from Laptops to Supercomputers” SoftwareX 1–2, (2015): 19–25.

[45]	W. L. Jorgensen, J. Chandrasekhar, J. D. Madura, R. W. Impey, M. L. Klein, “Comparison of Simple Potential Functions for Simulating Liquid Water” Journal of Chemical Physics 79, (1983): 926–935.

[46]	T. Darden, D. York, L. Pedersen, “Particle Mesh Ewald: An N·log(N) Method for Ewald Sums in Large Systems” Journal of Chemical Physics 98, (1993): 10089–10092.

[47]	B. Hess, H. Bekker, H. J. C. Berendsen, J. G. E. M. Fraaije, “LINCS: A Linear Constraint Solver for Molecular Simulations” Journal of Computational Chemistry 18, (1997): 1463–1472.

[48]	G. Bussi, D. Donadio, M. Parrinello, “Canonical Sampling through Velocity Rescaling” Journal of Chemical Physics 126, (2007): 014101.

[49]	M. Parrinello, A. Rahman, “Polymorphic Transitions in Single Crystals: A New Molecular Dynamics Method” Journal of Applied Physics 52, (1981): 7182–7190.

[50]	N. Michaud-Agrawal, E. J. Denning, T. B. Woolf, O. Beckstein, “MDAnalysis: A Toolkit for the Analysis of Molecular Dynamics Simulations” Journal of Computational Chemistry 32, (2011): 2319–2327.

[51]	R. J. Gowers, M. Linke, J. Barnoud, T. J. E. Reddy, M. N. Melo, S. L. Seyler, J. Domanski, D. L. Dotson, S. Buchoux, I. M. Kenney, et al., “MDAnalysis: A Python Package for the Rapid Analysis of Molecular Dynamics Simulations” (2019).

[52]	R. T. McGibbon, K. A. Beauchamp, M. P. Harrigan, C. Klein, J. M. Swails, C. X. Hernandez, C. R. Schwantes, L. P. Wang, T. J. Lane, V. S. Pande, “MDTraj: A Modern Open Library for the Analysis of Molecular Dynamics Trajectories” Biophysical Journal 109, (2015): 1528–1532.

[53]	W. Kabsch, C. Sander, “Dictionary of Protein Secondary Structure: Pattern Recognition of Hydrogen-Bonded and Geometrical Features” Biopolymers 22, (1983): 2577–2637.

[54]	J. Aguiar, P. Carpena, J. A. Molina-Bolívar, C. Carnero Ruiz, “On the Determination of the Critical Micelle Concentration by the Pyrene 1:3 Ratio Method” Journal of Colloid and Interface Science 258, (2003): 116–122.

[55]	Ö. Topel, B. A. Çakır, L. Budama, N. Hoda, “Determination of Critical Micelle Concentration of Polybutadiene-block-poly(ethyleneoxide) Diblock Copolymer by Fluorescence Spectroscopy and Dynamic Light Scattering” Journal of Molecular Liquids 177, (2013): 40–43.

[56]	Y. Kerkhoff, S. Wedepohl, C. Nie, V. Ahmadi, R. Haag, S. Block, “A Fast Open-Source Fiji-Macro to Quantify Virus Infection and Transfection on Single-Cell Level by Fluorescence Microscopy” MethodsX 9, (2022): 101834.

[57]	S. Y. van der Zanden, X. Qiao, J. Neefjes, “New Insights into the Activities and Toxicities of the Old Anticancer Drug Doxorubicin” FEBS Journal 288, (2021): 6095–6111
